# Case-control studies of gene-environment interactions. When a case might not be the case

**DOI:** 10.1371/journal.pone.0201140

**Published:** 2018-08-22

**Authors:** Iryna Lobach, Joshua Sampson, Alexander Alekseyenko, Siarhei Lobach, Li Zhang

**Affiliations:** 1 Department of Epidemiology and Biostatistics, University of California, San Francisco, San Francisco, California, United States of America; 2 National Cancer Institute, National Institutes of Health, Bethesda, MD, United States of America; 3 Department of Public Health Sciences, Medical University of South Carolina, Charleston, SC, United States of America; 4 Applied Mathematics and Computer Science Department, Belarusian State University, Minsk, Belarus; 5 Department of Medicine, University of California San Francisco, San Francisco, California, United States of America; 6 Helen Diller Family Comprehensive Cancer Center, University of California, San Francisco, San Francisco, California, United States of America; Torrey Pines Institute for Molecular Studies, UNITED STATES

## Abstract

Case-control Genome-Wide Association Studies (GWAS) provide a rich resource for studying the genetic architecture of complex diseases. A key is to elucidate how the genetic effects vary by the environment, what is traditionally defined by Gene-Environment interactions (GxE). The overlooked complication is that multiple, distinct pathophysiologic mechanisms may lead to the same clinical diagnosis and often these mechanisms have distinct genetic bases. In this paper, we first show that using the clinically diagnosed status can lead to severely biased estimates of GxE interactions in situations when the frequency of the pathologic diagnosis of interest, as compared to other diagnoses, depends on the environment. We then propose a pseudo-likelihood solution to correct the bias. Finally, we demonstrate our method in extensive simulations and in a GWAS of Alzheimer’s disease.

## Introduction

We are interested in using data from a case-control Genome-Wide Association Studies (GWAS) to estimate how an “environmental variable” modifies the effect of a genetic variant on a specific, pathologically defined disease state. However, the complication is that in many GWAS, the cases are a heterogenous group, where multiple distinct pathologically defined disease states have led to a common set of symptoms and a shared clinical diagnosis. In these scenarios, a genetic variant will appear to interact with the environmental variable if the genetic variant affects the pathologically defined disease state of interest and the environmental variable is related to the proportion of cases with that disease state.

The issue of heterogeneity among cases is, perhaps, most pronounced in neurologic and psychiatric disorders, where the clinically defined status is based primarily on descriptive criteria and is typically made in absence of biomarker measurements, imaging data, and biopsies. Our specific motivating study is a GWAS of late-onset Alzheimer’s disease (AD), a neurodegenerative disorder that is clinically characterized by progressive mental decline. Here, we are interested in identifying genetic variants specifically associated with a high abundance of amyloid deposits and neurofibrially tangles in the brain, which we refer to as “histopathologically defined AD.”[1] Specifically, we are interested in whether carrying the ApoE *ε*4 variant, which in the study is considered the “environmental variable”, modifies the effect of SNPs residing in Toll-Like Receptors (TLR) and Receptor for advanced glycation end products (RAGE) on histopathologically defined AD. Importantly, ApoE *ε*4 status is likely to be associated with the proportion of the GWAS cases who have histopathologically defined AD. Recent biomarker studies of AD [2] reported that 36% of ApoE *ε*4 non-carriers and 6% in ApoE *ε*4 carriers clinically diagnosed with AD do not have evidence of amyloid deposition. We provide a more detailed description of ApoE *ε*4, other the risk factors for AD and the heterogeneity of the disease in the Discussion section.

We are interested to test an association between single nucleotide polymorphisms (SNPs) residing in Toll-Like Receptors (TLR) and the true AD diagnosis, i.e. our goal is to identify the genetic that might have lea to amyloid plaques with associated cognitive decline. TLRs play a key role in an innate immune response to invading pathogens and are also important for triggering the adaptive immune responses. Dysregulation of human toll-like receptor function has been shown in aging [3]. Specifically to the etiology of AD, TLRs act through modification of the inflammatory state of microglia/macrophages [1]. Receptor for advanced glycation end products (RAGE) has been identified as receptor for amyloid-beta peptide [4].

There is an extensive literature on how the estimates of the main genetic effect can be biased in situations when disease status is misclassified, i.e. the clinical and pathologic diagnoses do not correspond [5]. We extend the literature by investigating the impact of misdiagnosis on estimates of the Gene-Environment interaction (GxE). In case-control studies, the effects of covariates have been traditionally assessed using logistic regression analysis [6]. Recently, however, Chatterjee and Carroll [7] noticed and proved that the assumptions of Hardy-Weinberg Equilibrium and Gene x Environment independence can be leveraged in the appropriate retrospective analyses to gain statistical efficiency. We adopt the principals derived by Chatterjee and Carroll [7] and develop a pseudo-likelihood model in settings when a case defined based on the clinical diagnosis might not be the case in terms of the *true* diagnosis defined pathophysiologically.

Our paper is organized as follows. First, in the Material and Methods section we present the setting, notation, and proposed pseudo-likelihood approach. Next, the Simulation Experiments section describes the simulation experiments conducted to compare the resulting performance of the proposed method with the performance of standard logistic regression using clinically defined disease. In the same section, we apply our method to the motivating study of AD. The Discussion section concludes the paper.

## Materials and methods

We define *G* be the genotype, e.g. SNPs measured at multiple locations. Let *X* be the environmental variables that interact with *G* and let *Z* be other environmental variables. We assume that the genotype is independent of all environmental variables and the genotypes follows Hardy-Weinberg Equilibrium: *G*~*Q*(*g*,*θ*). If *θ* is the frequency of minor allele a when the major allele is A, then the Hardy-Weinberg Equilibrium model [8] according to the number of minor alleles is

$$Pr(G=g|\theta )=\left\{ \begin{array}{c} 2\times \theta \times (1-\theta ),if\;g=Aa\\ \theta^{2},\;if\;g=aa\\ (1-\theta {)}^{2},\;if\;g=AA \end{array}\right.$$

We define *D*^*CL*^ = {0, 1} be observed clinical disease status defined based on a set of symptoms. Suppose that the same set of symptoms can be caused by two distinct pathophysiologic mechanisms. Let *D* be the *true* disease status defined based on the underlying pathology, where *D* = 1 indicates the disease of interest, while *D* = 1* is the nuisance disease. For ethical and/or budgetary reasons it might not be possible to measure the underlying pathology, hence *D* is latent. Instead, an evaluation is performed on a subset of patients or in an external reliability study. We define *τ*(*X*) = pr(*D* = 1|*D*^*CL*^ = 1,*X*) to be the frequency of the *true* diagnosis of interest within the clinically diagnosed set that varies by the environment *X*. We let probabilities of the clinical and *true* diagnoses in the population to be $\pi_{d^{cl}}=pr(D^{CL}=d^{cl})$ and *π*_*d*_ = pr(*D* =*d*), respectively.

The clinical and *true* diagnoses are related $pr(D^{CL}=d^{cl})=\sum_{d^{∎}}pr(D^{CL}=d^{cl}|D=d^{∎})\times pr(D=d^{∎})$, which indicates that the probabilities of the clinical diagnosis are weighted sums of frequencies of the *true* diagnoses. If pr(*D*^*CL*^ = *d*^*cl*^|*D* = *d*,*X* = *x*,*G* = *g*) = pr(*D*^*CL*^ = *d*^*cl*^|*D* =*d*), then *D*^*CL*^ is a *surrogate* of *D*. In this setting, $pr(D^{CL}=d^{cl}|G,X)=\sum_{d^{∎}}pr(D^{CL}=d^{cl}|D=d^{∎})\times pr(D=d^{∎}|X,G)$; hence if there is no relationship between (*X*,*G*) and *D*, neither there is one between (*X*,*G*) and *D*^*CL*^.

We first consider a binary setting where the risk parameters are defined in terms of *D* = 1 vs. *D* = 1* and *D* = 0 combined. Then the risk model is defined in terms of coefficients B = (*β*_0,_*β*_*G*_,*β*_*X*_,*β*_*Z*_,*β*_*G*×*X*_) by

$$log\left\{\frac{pr(D=1|G=g,X=x,Z=z)}{pr(D=1^{*}\;or\;0|G=g,X=x,Z=z)}\right\}=\beta_{0}+\beta_{G}\times g+\beta_{X}\times x+\beta_{Z}\times z+\beta_{G\times X}\times g\times x.$$ (1)

In the second setting that we consider the risk model is defined separately for *D* = 1 vs. *D* = 0 in terms of B = (*β*_0,_*β*_*G*_,*β*_*X*_,*β*_*Z*_,*β*_*G*×*X*_) and for *D* = 1* vs. *D* = 0 in terms of ${Β}^{*}=(\beta_{0}^{*},\beta_{G}^{*},\beta_{X}^{*},\beta_{Z}^{*},\beta_{G\times X}^{*})$ by

$$log\left\{\frac{pr(D=1|G=g,X=x,Z=z)}{pr(D=0|G=g,X=x,Z=z)}\right\}=\beta_{0}+\beta_{G}\times g+\beta_{X}\times x+\beta_{Z}\times z+\beta_{G\times X}\times g\times x;$$

$$log\left\{\frac{pr(D=1|G=g,X=x,Z=z)}{pr(D=1^{*}|G=g,X=x,Z=z)}\right\}=\beta_{0}^{*}+\beta_{0}^{*}\times g+\beta_{X}^{*}\times x+\beta_{Z}^{*}\times z+\beta_{G\times X}^{*}\times g\times x$$ (2)

In Eq (2) B and B* might share coefficients, e.g. if $\beta_{Z}=\beta_{Z}^{*}$.

The observed data are collected using a case-control design where genetic and environmental variables are measured after the disease status is ascertained. However, the data will be analyzed as a random sample. To facilitate this analysis, we let *δ* = 1 be an indicator of selection into the study and consider the imaginary Bernoulli sampling with $pr(\delta =1|D^{CL}=d^{cl})\propto n_{d^{cl}}/\pi_{d^{cl}}$. Define $\kappa_{d^{cl}}=\beta_{0}+log(n_{d^{cl}}/\pi_{d^{cl}})$ and $\kappa_{d^{cl}}^{*}=\beta_{0}^{*}+\log\left(\frac{n_{d^{cl}}}{\pi_{d^{cl}}}\right)$ with a parameter set Ω = (*κ*_0_,*β*_0_,*β*_*G*_,*β*_*X*_,*β*_*Z*_,*β*_*G*×*X*_,*θ*) For model (1) we define

$$S(d,d^{cl},g,x,z;\Omega )=\frac{\exp\left[I\left(d=1\right)\times \left\{\kappa_{d^{cl}}+\beta_{G}\times g+\beta_{X}\times x+\beta_{Z}\times z+\beta_{G\times X}\times g\times x\right\}\right]}{1+\exp\left\{\beta_{0}+\beta_{G}\times g+\beta_{X}\times x+\beta_{Z}\times z+\beta_{G\times X}\times g\times x\right\}}\times Q\left(g;\theta \right),$$

and for model (2) we define $\Omega =(\kappa_{0},\beta_{0},\beta_{G},\beta_{X},\beta_{Z},\beta_{G\times X},\beta_{0}^{*},\beta_{G}^{*},\beta_{X}^{*},\beta_{Z}^{*},\beta_{G\times X}^{*},\theta ).$

$$S(d,d^{cl},g,x,z;\Omega )=\frac{I\left(d=1\right)\times \exp\left\{\kappa_{d^{cl}}+\beta_{G}\times g+\beta_{X}\times x+\beta_{Z}\times z+\beta_{G\times X}\times g\times x\right\}+I\left(d=1^{*}\right)\times \exp\left\{\kappa_{d^{cl}}^{*}+\beta_{0}^{*}\times g+\beta_{X}^{*}\times x+\beta_{Z}^{*}\times z+\beta_{G\times X}^{*}\times g\times x\right\}}{1+\exp\left\{\beta_{0}+\beta_{G}\times g+\beta_{X}\times x+\beta_{Z}\times z+\beta_{G\times X}\times g\times x\right\}+\exp\left\{\beta_{0}^{*}+\beta_{0}^{*}\times g+\beta_{X}^{*}\times x+\beta_{Z}^{*}\times z+\beta_{G\times X}^{*}\times g\times x\right\}}\times Q\left(g;\theta \right).$$

In addition we let $\gamma_{d^{cl}|d}(X)=pr(D^{CL}=d^{cl}|D=d,X).$

Consider probability, Pr(*D*^*CL*^,*G*|*X*,*Z*,*δ* = 1) and define a function *L*(*d*^*CL*^,*g*,*x*,*z*;Ω) as follows.

$$L(d^{cl},g,x,z;\Omega )=\frac{S\left(0,0,g,x,z;\Omega \right)+\gamma_{d^{cl}|1}\left(g\right)\times S\left(1,d^{cl},g,x,z;\Omega \right)}{\sum_{g^{∎},d^{cl∎}}\left\{S\left(0,0,g^{∎},x,z;\Omega \right)+\gamma_{d^{cl∎}|1}\left(g^{∎}\right)\times S\left(1,d^{cl∎},g^{∎},x,z;\Omega \right)\right\}}.$$ (3)

The pseudo-likelihood

$$\prod_{i=1}^{N}L(d_{i}^{cl},g_{i},x_{i},z_{i};\Omega )$$ (4)

can be used in place of the likelihood function based on arguments provided in the Appendix. Define Ψ(*d*^*cl*^,*g*,*x*,*z*;Ω) to be the derivative of log{*L*(*d*^*cl*^,*g*,*x*,*z*;Ω)} with respect to Ω and

$$L_{N}(\Omega )=\sum_{i=1}^{N}\Psi (D_{i}^{CL},G_{i},X_{i},Z_{i};\Omega );$$

$$I=n^{-1}E\{\frac{\partial L_{N}(\Omega )}{\partial \Omega }\};$$

$$\Lambda =\sum_{d^{cl}}\frac{n_{d^{cl}}}{n}E\{\Psi (D_{i}^{CL},G_{i},X_{i},Z_{i};\Omega )|D^{CL}=d^{cl}\}\times {E\{\Psi (D_{i}^{CL},G_{i},X_{i},Z_{i};\Omega )|D^{CL}=d^{cl}\}}^{T},$$

where all expectations are taken with respect to the actual retrospective sampling scheme. Derivations shown in the Appendix demonstrate that under suitable regularity conditions there is a consistent sequence of solutions to $L_{n}(\Omega )=0$ with the following property

$$n^{\frac{1}{2}}(\hat{\Omega }-\Omega )⟹Normal\{0,I^{-1}(I-\Lambda )I^{-1}\}.$$

Remark 1: The intercept parameter $\kappa_{d^{cl}}$ is a function of the probability of disease in the population. Hence, if the probability of clinical diagnosis in the population is known or a good bound can be specified, this information can be used while estimating parameters. This cannot be done in the usual logistic regression setting.

## Results

### Simulation experiments

The goal of the simulation study is to examine potential differences in the effect estimates of the genetic and environmental variables in their relationship to the 1) observed clinical diagnosis using the usual logistic regression model (uLR) and pseudo-likelihood model (pMLE) [7]; and 2) to the *true* disease status by using our pseudo-likelihood approach (pMLE-DX) that takes into account that only a proportion of the clinically diagnosed cases have the true disease. In pMLE-DX parameters are estimated based on Eq (4). Parameters are compared by their Bias and Root Mean Squared Error (RMSE). Simulations are performed using MatLab version R2017a.

In each setting we simulate 500 datasets with *n*_0_ = *n*_1_ ∈ {1000,3000,5000,10000,50000}. We let the genotype (*G*) be a Bernoulli random variable with frequency 0.10 to mimic a SNP and allow its effect to follow a recessive or dominant model. We set our other parameters to be similar to the values observed in our GWAS of AD. The binary variable X = {*ε*4+,*ε*4−}, which represents the ApoE *ε*4 status according to presence or absence of *ε*4 allele that occurs in approximately 14% of the population.

The proportion of the nuisance disease within the clinical diagnosis is defined as pr(D = 1*|*D*^*CL*^ = 1,*ε*4−) = 0.36 and pr(D = 1*|*D*^*cl*^ = 1,*ε*4+) = 0.06. The clinical diagnosis of late onset AD is defined for ages 65 and older. We simulated age (*Z*_1_) to be Bernoulli with frequency 0.50 e.g. corresponding to a median split. Sex (*Z*_2_) is Bernoulli with frequency 0.52 to reflect what we observed in the motivating data example of AD.

#### Setting A

We first examine a setting when the nuisance disease and controls are equivalent in that the risk parameters are defined for the disease of interest vs. the combination of controls and nuisance disease as in Eq (1). The risk coefficients are $\beta_{0}=-1,\beta_{G}=0.406,\beta_{Z_{2}}=-0.083,\beta_{\varepsilon 4}=2.079,\beta_{G\times \varepsilon 4}=0.41$. In this setting, the frequency of the *true* disease status is pr(D = 1) = 46%, pr(D = 1|*ε*4−) = 40%, pr(D = 1|*ε*4+) = 82%. **Table 1** presents properties of the risk parameter estimates in the datasets with *n*_0_ = *n*_1_ = 3,000. Additionally, shown in **S1 Table** are studies with *n*_0_ = *n*_1_ ∈ {1000,5000,10000,50000}. When the presence of the nuisance disease is ignored (uLR, pMLE), ${\hat{\beta }}_{\varepsilon 4}$ and ${\hat{\beta }}_{G\times \varepsilon 4}$ are biased with elevated RMSE. For example, in a study with *n*_0_ = *n*_1_ = 3,000, the bias in ${\hat{\beta }}_{\varepsilon 4}$ is -0.31 in uLR and pMLE, while the bias is reduced to 0.005 by pMLE-DX. RMSE is 0.33 in uLR and pMLE, while it is reduced to 0.12 by pMLE-DX. Similarly, bias in ${\hat{\beta }}_{G\times \varepsilon 4}$ is 0.56 in uLR and pMLE, while pMLE-DX reduces the bias by more than half. RMSE of ${\hat{\beta }}_{G\times \varepsilon 4}$ is 2.5x larger when the presence of the nuisance disease is ignored. Notably, estimates of $\beta_{Z_{1}}$ and $\beta_{Z_{2}}$ are biased in uLR and pMLE. When sample size increased, the uLR bias in ${\hat{\beta }}_{G\times \varepsilon 4}$ decreased, e.g. the bias is 0.08 in a study with *n*_0_ = *n*_1_ = 10,000; while the bias in ${\hat{\beta }}_{\varepsilon 4}$ persisted. Across all sample sizes, ${\hat{\beta }}_{G}$ is biased by approximately -0.13, whereas considering the nuisance disease nearly eliminated the bias, e.g. to -0.01 in a study with *n*_0_ = *n*_1_ = 1000.

**Table 1:** Bias and RMSE in parameter estimates when *β*_*G*×*ε*4_ ≠ 0.

Parameters	True value		Clinical disease status is the outcome				With consideration of clinical-pathological diagnoses relationship	
			Usual logistic regression		Pseudo-likelihood method (pMLE)		Pseudo-likelihood method (pMLE-DX)	
			Bias	RMSE	Bias	RMSE	Bias	RMSE
*n*_0_ = 3,000 and *n*_1_ = 3,000							
*β*_0_	-1	0.46	0.46	0.98	0.98	-0.0002	0.07
*β*_*G*_	0.406	-0.13	0.16	-0.13	0.16	-0.008	0.13
$\beta_{Z_{1}}$	1.098	-0.35	0.35	-0.35	0.35	0.003	0.08
$\beta_{Z_{2}}$	-0.083	0.02	0.06	0.02	0.06	-0.004	0.08
*β*_*ε*4_	2.079	-0.31	0.33	-0.31	0.33	0.005	0.12
*β*_*G*×*ε*4_	0.693	0.56	2.4	0.26	0.91	0.22	0.93
Pr(G = 1)		0.10			-0.0004	0.004	0.02	0.02

The Bias and Root Mean Squared Error (RMSE) in parameter estimates from simulations using the usual logistic regression with clinical diagnosis as the outcome (uLR), the pseudo-likelihood approach (pMLE), and our newly proposed pseudo-likelihood approach that accounts for misdiagnosis (pMLE-DX). For these simulations, the study included *n*_0_ = 3000 controls and *n*_1_ = 3000 cases. Frequency of ApoE *ε*4 allele in the population is 14%. Variables *Z*_1_ and *Z*_2_ are Bernoulli with frequencies 0.50 and 0.52, respectively. Frequency of the *true* disease status is 46% in the population; and is 40% among the subpopulation with no ApoE *ε*4 alleles, and 82% in the subpopulation with at least one ApoE *ε*4 alleles. Frequency of nuisance disease within the clinical diagnosis varies by ApoE4 status pr(D = 1*|*D*^*CL*^ = 1,*ε*4−) = 0.36 and pr(D = 1*|*D*^*CL*^ = 1,*ε*4+) = 0.06.

We next examine if the presence of the nuisance disease could lead us to erroneously conclude that there was a significant ${\hat{\beta }}_{G\times \varepsilon 4}$ when *β*_*G*×*ε*4_ = 0. Here, we simulated datasets with *β*_*G*×*ε*4_ = 0. **Table 2** presents estimates in a study with *n*_0_ = *n*_1_ = 3000 and **S2 Table** is based on studies with *n*_0_ = *n*_1_ ∈ {1000,5000,10000,50000}. Estimates of $\beta_{0},\beta_{G},\beta_{Z_{1}},\beta_{\varepsilon 4},$ and *β*_*G*×*ε*4_ are clearly biased when the presence of the nuisance disease is ignored. For example, in a study with *n*_0_ = *n*_1_ = 3,000, pMLE-DX decreased the bias in ${\hat{\beta }}_{G\times \varepsilon 4}$ from 0.12 in uLR and pMLE to 0.04, while RMSE remained approximately the same 0.41 vs. 0.43. Similarly, pMLE-DX reduced the bias in ${\hat{\beta }}_{\varepsilon 4}$ from -0.26 in uLR to 0.007. At the same time, the RMSE of ${\hat{\beta }}_{\varepsilon 4}$ went from 0.28 (uLR, pMLE) to 0.12 (pMLE-DX). Increasing the sample size reduced the uLR bias for ${\hat{\beta }}_{G\times \varepsilon 4},$ e.g. the bias is 0.09 in a study with *n*_0_ = *n*_1_ = 10,000 but did not alleviate the substantial uLR bias in *β*_*ε*4_. Across all sample sizes considered, the uLR estimates of *β*_*G*_ are biased by approximately -0.12, while pMLE-DX reduced the bias to e.g. 0.01 in a study with 1,000 cases and 1,000 controls.

**Table 2:** Bias and RMSE in parameter estimates when *β*_*G*×*ε*4_ = 0.

Parameters	True value	Clinical disease status is the outcome				With consideration of clinical-pathological relationship	
		Usual logistic regression		Pseudo-likelihood method		Pseudo-likelihood method	
		Bias	RMSE	Bias	RMSE	Bias	RMSE
*n*_0_ = 3,000 and *n*_1_ = 3,000							
*β*_0_	-1	0.45	0.45	0.93	0.93	-0.0004	0.07
*β*_*G*_	1.099	-0.12	0.15	-0.07	-0.15	0.002	0.13
$\beta_{Z_{1}}$	1.098	-0.33	0.34	-0.33	0.34	0.001	0.08
$\beta_{Z_{2}}$	-0.083	0.02	0.06	0.02	0.06	-0.003	0.08
*β*_*ε*4_	2.079	-0.26	0.28	-0.26	0.28	0.007	0.12
*β*_*G*×*ε*4_	0	0.12	0.41	0.13	0.41	0.04	0.43
Pr(G = 1)	0.10			-0.000	0.004	0.03	0.03

The Bias and Root Mean Squared Error (RMSE) in parameter estimates from simulations using the usual logistic regression with clinical diagnosis as the outcome (uLR), the pseudo-likelihood approach (pMLE), and our newly proposed pseudo-likelihood approach that accounts for misdiagnosis (pMLE-DX). For these simulations, the study included *n*_0_ = 3000 controls and *n*_1_ = 3000 cases. Frequency of ApoE *ε*4 allele in the population is 14%. Variables *Z*_1_ and *Z*_2_ are Bernoulli with frequencies 0.50 and 0.52, respectively. Frequency of the *true* disease status is 46% in the population; and is 40% among the subpopulation with no ApoE *ε*4 alleles, and 82% in the subpopulation with at least one ApoE *ε*4 alleles. Frequency of nuisance disease within the clinical diagnosis varies by ApoE4 status pr(D = 1*|*D*^*CL*^ = 1,*ε*4−) = 0.36 and pr(D = 1*|*D*^*CL*^ = 1,*ε*4+) = 0.06.

We next consider the effect of underestimating pr(D = 1*|*D*^*CL*^ = 1,*ε*4+) and pr(D = 1*|*D*^*CL*^ = 1,*ε*4−) in the pseudo-likelihood. Here, we simulate data using the parameters specified above, but, when fitting the pseudo-likelihood (**S3 Table**), set pr(D = 1*|*D*^*CL*^ = 1,*ε*4−) = 0.3 and pr(D = 1*|*D*^*CL*^ = 1,*ε*4+) = 0, i.e. underestimated by 6%. Naturally, this misspecification introduced bias in some of the estimates and hence increased RMSE. Estimates of *β*_*ε*4_ were generally affected more than the estimates of the other parameters. For example, in a study with 3,000 cases and 3,000 controls, bias in ${\hat{\beta }}_{\varepsilon 4}$ increased from 0.005 to -0.66 in pMLE-DX, while RMSE went from 0.12 to 0.67. In estimates of *β*_*G*×*ε*4_, the bias increased from 0.22 to 0.32, while RMSE went up from 0.93 to 0.94. The bias in ${\hat{\beta }}_{G}$ increased to -0.10 in a study with 3,000 cases and 3,000 controls, what has not reached the level of uLR where the bias is -0.12. Estimates of $\beta_{X_{2}}$ remained nearly unbiased with the same RMSE.

We next consider the effect of overestimating pr(D = 1*|*D*^*CL*^ = 1,*ε*4+) and pr(D = 1*|*D*^*CL*^ = 1,*ε*4−) in the pseudo-likelihood (**S4 Table**). Here, we simulate data using the parameters specified above, but, when fitting the pseudo-likelihood, set pr(D = 1*|*D*^*CL*^ = 1,*ε*4−) = 0.42 and pr(D = 1*|*D*^*CL*^ = 1,*ε*4+) = 0.16, i.e. overestimated by 6%. As expected, this misspecification inflated the bias in the risk estimates. For example, in a study of 3,000 cases and 3,000 controls, bias in ${\hat{\beta }}_{\varepsilon 4}$ increased from 0.005 to -0.43, while RMSE went from 0.12 to 0.44. Bias in ${\hat{\beta }}_{G\times \varepsilon 4}$ decreased from 0.22 to 0.17, while RMSE remained the same. Estimates of *β*_*G*_ and $\beta_{X_{2}}$ remained nearly unbiased.

#### Setting B

We next examine a setting when two sets of parameters define the risk of disease, i.e. for *D* = 1 vs. *D* = 0 and *D* = 1* vs. *D* = 0 according to the risk model (2). **Table 3** (*n*_0_ = *n*_1_ = 3,000) and **S5 Table** present parameter estimates in the setting when $\beta_{0}=-1,\beta_{0}^{*}=-1.7,\beta_{G}=-0.69,\beta_{G}^{*}=0,\beta_{Z_{1}}=0.10,{\beta_{Z_{2}}=-0.083,\beta }_{\varepsilon 4}=1.3,\beta_{\varepsilon 4}^{*}=0.5,\beta_{G\times \varepsilon 4}=1.099,\beta_{G\times \varepsilon 4}^{*}=0,Pr(G=1)=0.$ With these parameters, the frequencies of the disease of interest and the nuisance disease are pr(*D* = 1) = 25.1%, pr(*D* = 1*) = 12.5%, pr(*D* = 1|*ε*4+) = 45.4%, pr(D = 1*|*ε*4+) = 16.1%, pr(D = 1|*ε*4−) =20%, pr(D = 1*|*ε*4−) = 16.1%. When presence of the nuisance disease is ignored (uLR, pMLE), estimates of *β*_0_,*β*_*ε*4_,*β*_*G*×*ε*4_,*β*_*G*_ are substantially biased.For example, in a study with 3,000 cases and 3,000 controls, in the bias of uLR for ${\hat{\beta }}_{\varepsilon 4}$ is -0.22, while pMLE-DX reduced this bias to -0.006; the bias of uLR for ${\hat{\beta }}_{G\times \varepsilon 4}$ is -0.13, while pMLE-DX reduced this bias to 0.01; the bias of uLR bias for ${\hat{\beta }}_{G}$ is 0.30, while pMLE-DX reduced it to 0.005. Biases in uLR persisted for larger sample sizes. If *a priori* evidence is sufficient to set parameters $\beta_{G\times \varepsilon 4}^{*}$ and $\beta_{G}^{*}$ to 0, when in fact these coefficients are zero, then RMSE of pMLE-DX are further reduced by at least 2-fold (data not shown).

**Table 3:** Bias and RMSE in parameter estimates when $\beta_{G}^{*}$ = 0 and $\beta_{G\times \varepsilon 4}^{*}$ = 0.

Parameters	True value	Clinical disease status is the outcome				With consideration of clinical-pathological diagnoses relationship	
		Usual logistic regression		Pseudo-likelihood method (pMLE)		Pseudo-likelihood method (pMLE-DX)	
		Bias	RMSE	Bias	RMSE	Bias	RMSE
*n*_0_ = 3,000 and *n*_1_ = 3,000							
*β*_0_	-1	0.97	0.97	0.74	0.74	0.02	0.06
$\beta_{0}^{*}$	-1.7					0.008	0.05
*β*_*G*_	-0.69	0.30	0.31	-0.39	0.39	0.005	0.10
$\beta_{G}^{*}$	0					-0.02	0.14
$\beta_{Z_{1}}$	0.10	0.002	0.31	0.004	0.05	0.002	0.05
$\beta_{Z_{2}}$	-0.083	-0.004	0.05	-0.0008	0.05	-0.004	0.05
*β*_*ε*4_	1.3	-0.22	0.24	-0.21	0.23	-0.006	0.10
$\beta_{\varepsilon 4}^{*}$	0.5					-0.007	0.05
*β*_*G*×*ε*4_	0.10	-0.13	0.29	-0.28	0.36	0.01	0.25
$\beta_{G\times \varepsilon 4}^{*}$	0					0.001	0.11
Pr(G = 1)	0.10			0.05	0.05	0.0001	0.004

The Bias and Root Mean Squared Error (RMSE) in parameter estimates from simulations using the usual logistic regression with clinical diagnosis as the outcome (uLR), the pseudo-likelihood approach (pMLE), and our newly proposed pseudo-likelihood approach that accounts for misdiagnosis (pMLE-DX). For these simulations, the study included *n*_0_ = 3000 controls and *n*_1_ = 3000 cases. Risk of the disease of interest is defined in a set of parameters $\beta_{0},\beta_{G},\beta_{Z_{1}},\beta_{Z_{2}},\beta_{G\times \varepsilon 4}$; while the risk of the nuisance disease is parametrized by $\beta_{0}^{*},\beta_{G}^{*},\beta_{\varepsilon 4}^{*},\beta_{G\times \varepsilon 4}^{*}.$ Frequency of ApoE *ε*4 allele in the population is 14%. Variables *Z*_1_ and *Z*_2_ are Bernoulli with frequencies 0.50 and 0.52, respectively. Frequencies of the disease of interest and the nuisance disease are pr(D = 1) = 24.8%, pr(D = 1*) = 12.5%, pr(D = 1|*ε*4+) = 43%, pr(D = 1*|*ε*4+) = 16.1%, pr(D = 1|*ε*4−) = 20%, pr(D = 1*|*ε*4+) = 11.6%. Frequency of the nuisance disease within the clinical diagnosis varies by ApoE4 status pr(D = 1*|*D*^*CL*^ = 1,*ε*4−) = 0.36 and pr(D = 1*|*D*^*CL*^ = 1,*ε*4+) = 0.06.

**Table 4** and **S6 Table** present the results in a setting similar to that of **Table 3** but when there is no interaction between the genotype and ApoE4 status, i.e. *β*_*G*×*ε*4_ = 0. Ignoring the nuisance disease in the uLR resulted in bias in the estimate of *β*_*G*×*ε*4_ that is -0.23, which might mislead to a conclusion that there is an interactive effect between the genotype and ApoE *ε*4 status. The bias persisted for larger sample sizes.

**Table 4:** Bias and RMSE in parameter estimates when $\beta_{G}^{*}$ = 0, *β*_*G*×*ε*4_ = 0 and $\beta_{G\times \varepsilon 4}^{*}$ = 0.

Parameters	True value	Clinical disease is the outcome				With consideration of clinical-pathological diagnoses relationship	
		Usual logistic regression		Pseudo-likelihood method (pMLE)		Pseudo-likelihood method (pMLE-DX)	
		Bias	RMSE	Bias	RMSE	Bias	RMSE
*n*_0_ = 3,000 and *n*_1_ = 3,000							
*β*_0_	-1	0.97	0.97	0.75	0.75	0.03	0.06
$\beta_{0}^{*}$	-1.7					0.01	0.05
*β*_*G*_	-0.69	0.30	0.31	-0.38	0.39	0.004	0.09
$\beta_{G}^{*}$	0					-0.01	0.13
$\beta_{Z_{1}}$	0.10	0.002	0.05	0.001	0.09	0.002	0.05
$\beta_{Z_{2}}$	-0.083	-0.004	0.05	-0.003	0.05	-0.004	0.05
*β*_*ε*4_	1.3	-0.22	0.24	-0.22	0.23	-0.006	0.10
$\beta_{\varepsilon 4}^{*}$	0.5					-0.009	0.06
*β*_*G*×*ε*4_	0	-0.23	0.28	-0.23	0.28	0.01	0.25
$\beta_{G\times \varepsilon 4}^{*}$	0					-0.0008	0.12
Pr(G = 1)	0.10					0.000	0.004

The Bias and Root Mean Squared Error (RMSE) in parameter estimates from simulations using the usual logistic regression with clinical diagnosis as the outcome (uLR), the pseudo-likelihood approach (pMLE), and our newly proposed pseudo-likelihood approach that accounts for misdiagnosis (pMLE-DX). For these simulations, the study included *n*_0_ = 3000 controls and *n*_1_ = 3000 cases. Risk of the disease of interest is defined in a set of parameters $\beta_{0},\beta_{G},\beta_{Z_{1}},\beta_{Z_{2}},\beta_{G\times \varepsilon 4}$; while the risk of the nuisance disease is parametrized by $\beta_{0}^{*},\beta_{G}^{*},\beta_{\varepsilon 4}^{*},\beta_{G\times \varepsilon 4}^{*}.$ Frequency of ApoE *ε*4 allele in the population is 14%. Variables *Z*_1_ and *Z*_2_ are Bernoulli with frequencies 0.50 and 0.52, respectively. Frequencies of the disease of interest and the nuisance disease are pr(D = 1) = 24.8%, pr(D = 1*) = 12.5%, pr(D = 1|*ε*4+) = 43%, pr(D = 1*|*ε*4+) = 16.1%, pr(D = 1|*ε*4−) = 20%, pr(D = 1*|*ε*4−) = 11.6%. Frequency of the nuisance disease within the clinical diagnosis varies by ApoE4 status pr(D = 1*|*D*^*CL*^ = 1,*ε*4−) = 0.36 and pr(D = 1*|*D*^*CL*^ = 1,*ε*4+) = 0.06.

#### Setting C

We next conducted a simulation study to better understand the underlying nature of the biases in the estimates noted when presence of the nuisance disease is ignored (uLR). For clarity, we simulated all variables to be binary. Variables *G*,*Z*_1_ and *Z*_2_ are Bernoulli with frequencies 0.10, 0.52 and 0.50, respectively. Risk coefficients are $\beta_{0}=-1,\beta_{G}=\log(1.5)=0.41,\beta_{Z_{1}}=1,\beta_{Z_{2}}=\log(0.92)=-0.08,\;\beta_{\varepsilon 4}=\log(8)=2.1,\beta_{G\times \varepsilon 4}=\log(3)=1.1.$ Then we varied values of $\beta_{X_{2}},\beta_{\varepsilon 4}$, and *β*_*G*×*ε*4_. The relationship between clinical and pathophysiological diagnosis is set to be pr(D = 1*|*D*^*CL*^ = 1,*ε*4−) = 0.36 and pr(D = 1*|*D*^*CL*^ = 1,*ε*4+) = 0.06. We simulated 500 datasets with 3,000 cases and 3,000 controls.

**Fig 1:**
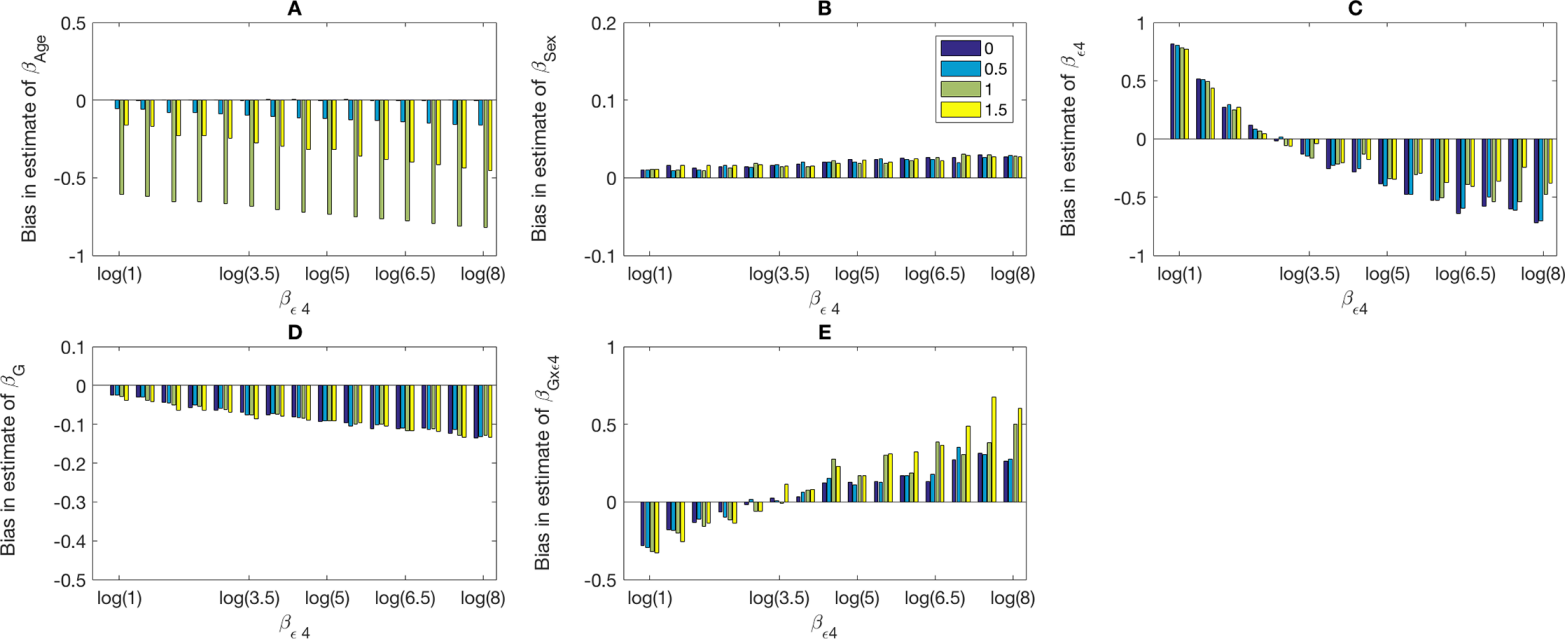
**The bias in estimates of** $\beta_{Z_{1}}$ **(*β*_*Age*_) (A)**, $\beta_{Z_{2}}$ **(*β*_*Sex*_) (B), *β*_*ε*4_ (C), *β*_*G*_ (D), and *β*_*G*×*ε*4_ (E) obtained using the usual logistic regression with clinical diagnosis as the outcome across values of *β*_*ε*4_**. Simulated are datasets with 3,000 cases and 3,000 controls. Values of *β*_*ApoE*4_ are listed along the x-axis and the true values of $\beta_{Z_{1}}$ are indicated by color. The parameters are set as follows: *β*_0_ = −1, *β*_*G*_ = log(1.5), $\beta_{Z_{2}}=-0.083,$ *β*_*G*×*ε*4_ = log(3); the relationship between the clinical and *true* disease statuses is pr(D = 1*|*D*^*CL*^ = 1,*ε*4-) = 0.36 and pr(D = 1*|*D*^*CL*^ = 1,*ε*4+) = 0.06. Variables *G*,*Z*_1_ and *Z*_2_ are Bernoulli with frequencies 0.10, 0.50 and 0.52, respectively.

**Fig 1** presents a study where *β*_*ε*4_ varies as log(1),log(1.5),log(2),log(2.5),…log(8) across the x-axis and $\beta_{Z_{2}}$ is color-coded to be 0, 0.5, 1, 1.5. We show in panels A, B, C, D, and E, the biases of ${\hat{\beta }}_{Z_{2}},{\hat{\beta }}_{Z_{1}},{\hat{\beta }}_{\varepsilon 4},{\hat{\beta }}_{G}$, and ${\hat{\beta }}_{G\times \varepsilon 4}$, respectively. With increasing value of *β*_*ε*4_, the biases in the main effect estimates of $\beta_{Z_{2}},\beta_{Z_{1}}$ and *β*_*G*_ increase. For example, the bias in ${\hat{\beta }}_{G}$ reaches -0.10 when *β*_*ε*4_ is log(5). The bias in ${\hat{\beta }}_{\varepsilon 4}$ and ${\hat{\beta }}_{G\times \varepsilon 4}$ is even more sensitive to value of *β*_*ε*4_. For example, when *β*_*ε*4_ = 0, the bias in ${\hat{\beta }}_{G\times \varepsilon 4}$ is 0.8; while when *β*_*ε*4_ = log(8) the bias is -0.7. Similarly, when *β*_*ε*4_ = 0, the bias in ${\hat{\beta }}_{G\times \varepsilon 4}$ is -0.18; while when *β*_*ε*4_ = log(8) the bias becomes 0.6. Bias in the estimates of $\beta_{X_{2}}$ increases with the increase in the true value. Bias in the other estimates is nearly not affected by values of $\beta_{X_{2}}$.

**Fig 2:**
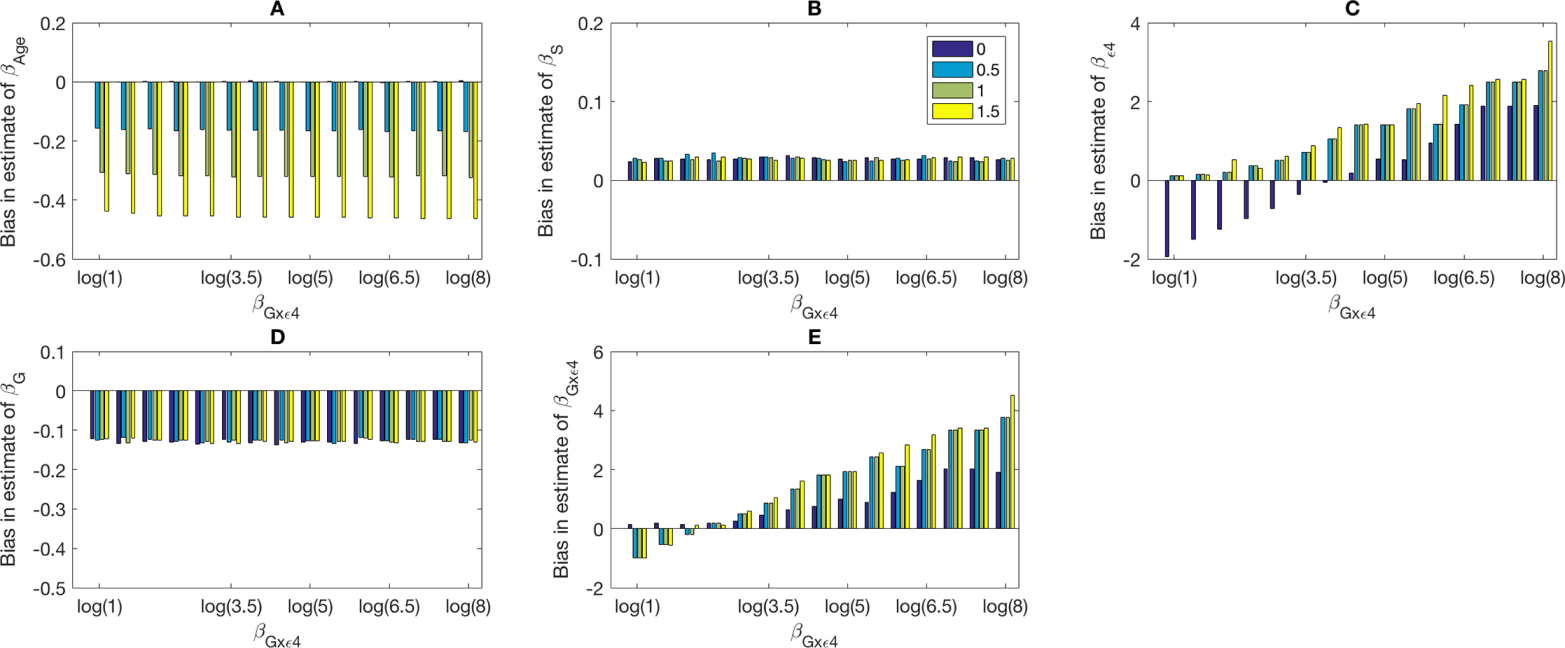
**The bias in estimates of** $\beta_{Z_{1}}$ **(*β*_*Age*_) (A),** $\beta_{Z_{2}}$ **(*β*_*Sex*_) (B), *β*_*ε*4_ (C), *β*_*G*_ (D), and *β*_*G*×*ε*4_ (E) obtained using the usual logistic regression with clinical diagnosis as the outcome across values of *β*_*G*×*ε*4_**. Simulated are datasets with 3,000 cases and 3,000 controls. Values of *β*_*G*×*ApoE*4_ are listed along the x-axis and the true values of $\beta_{Z_{1}}$ are indicated by color. The parameters are set as follows: *β*_0_ = −1, *β*_*G*_ = log(1.5), $\beta_{X_{2}}=-0.083,$ *β*_*G*×*ε*4_ = log(3); the relationship between the clinical and *true* disease statuses is pr(D = 1*|*D*^*CL*^ = 1,*ε*4-) = 0.36 and pr(D = 1*|*D*^*CL*^ = 1,*ε*4+) = 0.06. Variables *G*,*Z*_1_ and *Z*_2_ are Bernoulli with frequencies 0.10, 0.50 and 0.52, respectively.

**Fig 2** presents a study where *β*_*G*×*ε*4_ varies as log(1),log(1.5),log(2),log(2.5),…log(8) across the x-axis and $\beta_{Z_{2}}$ is color-coded to be 0, 0.5, 1, 1.5. We show in panels A, B, C, D, and E, the bias of ${\hat{\beta }}_{Z_{2}},\;{\hat{\beta }}_{Z_{1}},\;{\hat{\beta }}_{\varepsilon 4},\;{\hat{\beta }}_{G},\;{\hat{\beta }}_{G\times \varepsilon 4}$, respectively. In this setting, the biases in the main effects ${\hat{\beta }}_{Z_{2}},\;{\hat{\beta }}_{Z_{1}}$ and ${\hat{\beta }}_{G}$ were approximately the same for all values of *β*_*G*×*ε*4_, while the biases in the estimates of ${\hat{\beta }}_{\varepsilon 4}$ and ${\hat{\beta }}_{G\times \varepsilon 4}$ were more sensitive to the value of *β*_*G*×*ε*4_. For example, when the interaction coefficient is 0, the bias of ${\hat{\beta }}_{\varepsilon 4}$ is nearly -2, while when *β*_*G*×*ε*4_ = log(8) = 2.08, the bias goes up to 3. When *β*_*G*×*ε*4_ = 0, the bias in the estimate is nearly zero, while the bias goes to almost 6 when the true value is log(8).

### Analyses of genetic variants serving toll-like receptors and receptor for advanced glycation end products in Alzheimer’s disease

We applied the proposed analyses to a dataset collected as part of the Alzheimer’s Disease Genetics Consortium. The data has been anonymized prior to access by the authors. The data consists of 1,245 controls and 2,785 cases. The average age (SD) of Cases and controls are 72.1 (9.1) and 70.9 (8.8) years, respectively. Among cases, 1,458 (52.4%) are men; among controls, 678 (63.9%) are men. At least one ApoE *ε*4 allele is present in (64.5%) of cases and 365 (29.1%) of controls.

Illumina Human 660K markers have been mapped onto human chromosomes using NCBI dbSNP database (https://www.ncbi.nlm.nih.gov/projects/SNP/). Chromosome location, proximal gene or genes and gene structure location (e.g. intron, exon, intergenic, UTR) has been recorded for all SNPs. From these data, we inferred 111 SNPs to reside in genes serving Toll-Like Receptors (TLR). Similarly, we inferred 3 SNPs to reside in the Receptor for advanced glycation end products (AGER).

It is of interest to examine a relationship between the pathologic diagnosis and each of the 111 TLR SNPs (*G*), ApoE *ε*4 status (*X*), age (*Z*_1_), sex (*Z*_2_). The effect of SNPs might vary by ApoE *ε*4 hence we included interaction between the genotype and ApoE *ε*4 status. The genetic variables are modeled using a binary indicator of presence or absence of a minor allele.

We estimate parameters using the standard logistic model (uLR) that uses the clinical diagnosis as a surrogate of the pathophysiologic diagnosis and the pseudo-likelihood model (pMLE-DX) where we assume that the relationship between the clinical and pathophysiologic diagnosis is as estimated in the Salloway study [2], i.e. the proportion of the nuisance disease within the clinically diagnosed set is 36% in ApoE *ε*4 non-carriers and 6% in ApoE *ε*4 carriers. The pseudo-likelihood model pMLE-DX estimates the coefficients in a model that treats the nuisance disease and controls equivalently as in Eq (1). pMLE-DX*, however, estimates two sets of the risk coefficients as in Eq (2). Data analyses are performed using MatLab version R2017a. When optimizing the pseudolikelihood function we bounded the estimates to be on the interval [–5,5].

We first examine the results when statistical significance is assessed according to p-value<0.05. We next correct for false discovery rate using Benjanimi-Hochberg method [9].

#### TLR

Shown in **Table 5** are estimates of the risk coefficients for 53 SNPs with permutation-based p-values for ${\hat{\beta }}_{G}$ or ${\hat{\beta }}_{G\times \varepsilon 4}$ that are <0.05 in either of the analyses. Of these 53 SNPs, 28 SNPs are within 500k up- or downstream of the SNPs previously reported in GWAS on Alzheimer’ disease, dementia, tauopathy, or/and vascular disease (**S6 Table**).

**Table 5:** Parameter estimates in Alzheimer’s disease study.

**SNP**	**Gene/Intergenic Region**	**Method**	${\hat{\beta }}_{Age}$	${\hat{\beta }}_{Sex}$	${\hat{\beta }}_{\varepsilon 4}$	${\hat{\beta }}_{G}$	${\hat{\beta }}_{G\times \varepsilon 4}$
**rs2033831**	KIAA0922 | TLR2	uLR pMLE-DX pMLE-DX*	**-0.24**, p = 0.00 -1.6, p = 0.13 **-0.24**, p = 0.00	**-0.43**, p = 0.00 -2.4, p = 0.11 **-0.43**, p = 0.00	**0.86**, p = 0.00 -2.6, p = 0.32 1.9, p = 0.25	-0.30, p = 0.09 -0.87, p = 0.33 4.2, p = 0.28	**0.58**, p = 0.03 3.7, p = 0.80 0.22, p = 0.28
**rs7656500**	KIAA0922 | TLR2	uLR pMLE-DX pMLE-DX*	**-0.25**, p = 0.00 -1.2, p = 0.25 -0.25, p = 0.00	**-0.42**, p = 0.00 -1.8, p = 0.27 -0.43, p = 0.00	**1.6**, p = 0.01 0.12, p = 0.57 -2.3, p = 0.27	**0.79**, p = 0.01 **6.0**, p = 0.02 5.0, p = 0.13	-0.16, p = 0.43 0.73, p = 0.27 4.3, p = 0.09
**rs1816702**	TLR2	uLR pMLE-DX pMLE-DX*	**-0.24**, p = 0.00 -1.4, p = 0.13 -0.24, p = 0.00	**-0.43**, p = 0.00 -2.1, p = 0.10 -0.43, p = 0.00	**1.4**, p = 0.00 -0.18, p = 0.50 **2.6**, p = 0.03	0.43, p = 0.06 **5.0**, p = 0.04 5.0, p = 0.38	0.01, p = 0.49 1.1, p = 0.65 -0.46, p = 0.13
**rs830832**	SORBS2 | TLR3	uLR pMLE-DX pMLE-DX*	**-0.24**, p = 0.00 -1.0, p = 0.16 **-0.24**, p = 0.00	**-0.42**, p = 0.00 -1.6, p = 0.10 **-0.43**, p = 0.00	**0.74**, p = 0.01 -0.64, p = 0.42 -1.4, p = 0.07	-0.31, p = 0.06 -0.55, p = 0.35 4.4, p = 0.21	**0.74**, p = 0.01 1.8, p = 0.70 **2.6**, p = 0.03
**rs7676342**	SORBS2 | TLR3	uLR pMLE-DX pMLE-DX*	**-0.25**, p = 0.00 -1.3, p = 0.19 **-0.24**, p = 0.00	**-0.43**, p = 0.00 -2.2, p = 0.15 **-0.43**, p = 0.00	**1.6**, p = 0.00 0.94, p = 0.72 **1.5**, p = 0.03	**0.35**, p = 0.04 0.88, p = 0.75 4.3, p = 0.66	-0.24, p = 0.21 -0.02, p = 0.53 0.23, p = 0.69
**rs4862611**	SORBS2 | TLR3	uLR pMLE-DX pMLE-DX*	**-0.24**, p = 0.00 **-1.5**, p = 0.13 **-0.25**, p = 0.00	**-0.43**, p = 0.00 **-2.9**, p = 0.09 **-0.43**, p = 0.00	**1.4**, p = 0.00 0.04, p = 0.56 1.7, p = 0.08	0.08, p = 0.24 -0.36, p = 0.35 2.2, p = 0.15	0.03, p = 0.55 1.2, p = 0.32 **-2.8**, p = 0.03
**rs13113778**	SORBS2 | TLR3	uLR pMLE-DX pMLE-DX*	**-0.24**, p = 0.00 -1.7, p = 0.16 **-0.24**, p = 0.00	**-0.43**, p = 0.00 -4.9, p = 0.05 **-0.42**, p = 0.00	**2.0**, p = 0.00 4.1, p = 0.10 -0.75, p = 0.49	0.13, p = 0.62 -2.1, p = 0.26 5.0,p = 0.36	-0.64, p = 0.14 -3.1, p = 0.25 2.7, p = 0.06
**rs1869617**	SORBS2 | TLR3	uLR pMLE-DX pMLE-DX*	**-0.24**, p = 0.00 -1.4, p = 0.14 **-0.24**, p = 0.00	**-0.43**, p = 0.00 -2.7, p = 0.09 **-0.43**, p = 0.00	**0.96**, p = 0.02 -3.1, p = 0.32 **2.2**, p = 0.00	-0.33, p = 0.15 -0.53, p = 0.39 **4.6**, p = 0.01	0.46, p = 0.86 4.0, p = 0.11 0.18, p = 0.51
**rs11938703**	SORBS2 | TLR3	uLR pMLE-DX pMLE-DX*	**-0.24**, p = 0.002 -0.78, p = 0.15 **-0.25**, p = 0.00	**-0.42**, p = 0.00 -1.2, p = 0.13 **-0.42**, p = 0.00	**0.95**, p = 0.00 0.09, p = 0.57 1.7, p = 0.20	-0.24, p = 0.05 -0.50, p = 0.28 2.8, p = 0.28	**0.58**, p = 0.00 1.2, p = 0.67 -0.57, p = 0.35
**rs1519318**	SORBS2 | TLR3	uLR pMLE-DX pMLE-DX*	**-0.24**, p = 0.00 -1.4, p = 0.14 **-0.25**, p = 0.00	**-0.43**, p = 0.00 -2.6, p = 0.10 **-0.43**, p = 0.00	**1.2**, p = 0.00 0.86, p = 0.70 2.1, p = 0.08	-0.01,p = 0.51 0.04, p = 0.59 **3.3**, p = 0.01	0.21, p = 0.16 0.07, p = 0.44 -0.41, p = 0.39
**rs12648771**	SORBS2 | TLR3	uLR pMLE-DX pMLE-DX*	**-0.25**, p = 0.00 -1.6, p = 0.15 -0.24, p = 0.00	**-0.42**, p = 0.00 -2.0, p = 0.13 -0.43, p = 0.00	**3.0**, p = 0.00 2.9, p = 0.15 0.62, p = 0.06	-0.27, p = 0.26 -1.6, p = 0.26 5.0, p = 0.24	**-1.6**, p = 0.004 -2.1, p = 0.38 0.02, p = 0.31
**rs6894**	NQO1 | LOC100132364	uLR pMLE-DX pMLE-DX*	**-0.24**, p = 0.00 -1.4, p = 0.12 -0.24, p = 0.00	**-0.43**, p = 0.00 -2.4, p = 0.09 -0.42, p = 0.00	0.59, p = 0.07 0.42, p = 0.64 **2.8**, p = 0.00	**-0.64**, p = 0.03 -0.20, p = 0.46 **3.9**, p = 0.00	**0.84**, p = 0.03 0.49, p = 0.59 0.84, p = 0.16
**rs3775296**	TLR3	uLR pMLE-DX pMLE-DX*	**-0.24**, p = 0.00 -1.4, p = 0.14 **-0.24**, p = 0.00	**-0.43**, p = 0.00 -2.4, p = 0.11 **-0.42**, p = 0.00	**2.0**, p = 0.00 3.4, p = 0.14 **1.4**, p = 0.02	-0.02, p = 0.50 0.81, p = 0.27 **4.3**, p = 0.046	-0.61, p = 0.06 -2.5, p = 0.33 0.89, p = 0.12
**rs7668666**	TLR3	uLR pMLE-DX pMLE-DX*	**-0.24**, p = 0.00 -1.7, p = 0.13 **-0.24**, p = 0.00	**-0.43**, p = 0.00 **-4.9**, p = 0.018 **-0.42**, p = 0.00	**1.4**, p = 0.00 3.4, p = 0.14 **1.7**, p = 0.02	-0.14, p = 0.25 1.7, p = 0.19 **4.9**, p = 0.00	0.01, p = 0.52 -2.6, p = 0.27 -0.81, p = 0.19
**rs1706143**	TLR3 | FAM149A	uLR pMLE-DX pMLE-DX*	**-0.24**, p = 0.00 -1.4, p = 0.14 **-0.24**, p = 0.00	**-0.43**, p = 0.00 -2.4, p = 0.11 **-0.42**, p = 0.00	**1.3**, p = 0.00 0.33, p = 0.37 0.45, p = 0.67	-0.12, p = 0.20 -0.39, p = 0.32 3.8, p = 0.26	0.12, p = 0.26 0.66, p = 0.33 **2.9**, p = 0.03
**rs9299251**	ASTN2 | TLR4	uLR pMLE-DX pMLE-DX*	**-0.25**, 0.00 -1.3, p = 0.14 -0.24, p = 0.00	**-0.43**, p = 0.00 -2.1, p = 0.14 -0.42, p = 0.00	**1.1**, p = 0.00 0.00, p = 0.63 0.48, p = 0.18	-0.04, p = 0.65 0.19, p = 0.65 3.3, p = 0.45	**0.35**, p = 0.04 1.1, p = 0.32 0.47, p = 0.18
**rs955302**	TNFRSF19	uLR pMLE-DX pMLE-DX*	**-0.24**, p = 0.00 -1.5, p = 0.13 **-0.25**, p = 0.00	**-0.43**, p = 0.00 -2.5, p = 0.10 **-0.43**, p = 0.00	**1.2**, p = 0.00 1.2, p = 0.34 **2.2**, p = 0.02	-0.28, p = 0.06 -0.23, p = 0.40 3.9, p = 0.30	0.27, p = 0.13 -0.37, p = 0.44 -1.2, p = 0.16
**rs17419570**	ASTN2 | TLR4	uLR pMLE-DX pMLE-DX*	**-0.24**, p = 0.00 -0.91, p = 0.23 **-0.24**, p = 0.00	**-0.44**, p = 0.00 -1.5, p = 0.21 **-0.42**, p = 0.00	0.68, p = 0.06 -0.93, p = 0.50 1.2, p = 0.44	**-0.94**, p = 0.01 -1.8, p = 0.32 **4.5**, p = 0.008	0.74, p = 0.06 1.9, p = 0.2 1.6, p = 0.064
**rs16905625**	ASTN2 | TLR4	uLR pMLE-DX pMLE-DX*	**-0.24**, p = 0.00 -1.5, p = 0.19 **-0.25**, p = 0.00	**-0.43**, p = 0.00 -3.3, p = 0.13 **-0.43**, p = 0.00	**1.3**, p = 0.01 3.3, p = 0.12 0.92, p = 0.11	0.04, p = 0.59 2.3, p = 0.15 **3.2**, p = 0.03	0.16, p = 0.69 -2.5, p = 0.28 -0.33,p = 0.32
**rs10513307**	ASTN2 | TLR4	uLR pMLE-DX pMLE-DX*	**-0.24**, p = 0.00 -1.7, p = 0.15 **-0.24**, p = 0.00	**-0.43**, p = 0.00 **-5.0**, p = 0.03 **-0.43**,p = 0.00	**1.7**, p = 0.01 3.9, p = 0.11 -1.3, p = 0.33	0.06, p = 0.56 -0.61, p = 0.41 **5.0**, p = 0.02	-0.28, p = 0.31 -3.0, p = 0.32 2.5, p = 0.09
**rs1890047**	ASTN2 | TLR4	uLR pMLE-DX pMLE-DX*	**-0.25**, p = 0.002 -1.3, p = 0.11 **-0.25**, p = 0.00	**-0.43**, p = 0.00 -2.2, p = 0.10 **-0.43**, p = 0.00	**1.1**, p = 0.00 -0.77, p = 0.45 -0.17, p = 0.44	-0.06, p = 0.34 -0.08, p = 0.44 **4.6**, p = 0.008	**0.39**,p = 0.02 1.9, p = 0.22 2.0, p = 0.064
**rs4837254**	ASTN2 | TLR4	uLR pMLE-DX pMLE-DX*	**-0.24**, p = 0.00 -1.7, p = 0.15 **-0.24**, p = 0.00	**-0.43**, p = 0.00 **-5.0**, p = 0.03 **-0.42**, p = 0.00	**1.2**, p = 0.00 3.9, p = 0.11 **2.0**, p = 0.04	-0.03, p = 0.43 -0.61, p = 0.41 3.4, p = 0.65	0.27, p = 0.08 -3.0, p = 0.32 -0.90, p = 0.25
**rs13285674**	ASTN2 | TLR4	uLR pMLE-DX pMLE-DX*	**-0.24**, p = 0.002 -1.6, p = 0.14 **-0.24**, p = 0.00	**-0.43**, p = 0.00 -3.1, p = 0.08 **-0.43**, p = 0.00	**1.0**, p = 0.00 0.31, p = 0.62 **2.8**, p = 0.00	**-0.47**, p = 0.003 -1.3, p = 0.29 **4.5**, p = 0.03	0.42, p = 0.10 0.63, p = 0.40 -0.65, p = 0.24
**rs1337208**	ASTN2 | TLR4	uLR pMLE-DX pMLE-DX*	**-0.24**, p = 0.00 -1.2, p = 0.12 **-0.25**, p = 0.00	**-0.43**, p = 0.00 -2.0, p = 0.10 **-0.42**, p = 0.00	**1.1**, p = 0.00 -0.03, p = 0.55 0.96, p = 0.79	-0.009, p = 0.49 0.15, p = 0.62 2.9, p = 0.15	**0.34**, p = 0.05 1.1, p = 0.31 0.46, p = 0.71
**rs1415378**	ASTN2 | TLR4	uLR pMLE-DX pMLE-DX*	**-0.25**, p = 0.00 -1.5, p = 0.14 **-0.24**, p = 0.00	**-0.43**, p = 0.00 -2.8, p = 0.10 **-0.42**, p = 0.00	**1.4**, p = 0.00 1.5, p = 0.20 0.50, p = 0.28	0.05, p = 0.32 0.98, p = 0.21 2.1, p = 0.06	0.05, p = 0.54 -0.62, p = 0.37 1.4, p = 0.08
**Rs504204**	ASTN2 | TLR4	uLR pMLE-DX pMLE-DX*	**-0.24**, p = 0.002 -1.5, p = 0.16 **-0.24**, p = 0.00	**-0.43**, p = 0.00 -2.9, p = 0.13 **-0.43**, p = 0.00	0.21, p = 0.63 -4.0, p = 0.27 **-3.9** p = 0.16	0.11, p = 0.54 -2.5, p = 0.48 5.0, p = 0.11	1.2, p = 0.17 **5.0**, p = 0.044 4.6, p = 0.06
**rs12337381**	ASTN2 | TLR4	uLR pMLE-DX pMLE-DX*	**-0.25**, p = 0.00 -1.5, p = 0.14 **-0.25,** p = 0.00	**-0.43**, p = 0.00 -2.5, p = 0.10 **-0.43,** p = 0.00	**0.84**, p = 0.04 -0.63, p = 0.50 **3.0**, p = 0.01	0.14, p = 0.66 -0.60, p = 0.40 4.6, p = 0.08	0.59, p = 0.11 1.6, p = 0.30 -1.5, p = 0.09
**rs1952464**	ASTN2 | TLR4	uLR pMLE-DX pMLE-DX*	**-0.25**, p = 0.00 -1.5, p = 0.15 **-0.25**, p = 0.00	**-0.43**, p = 0.00 -2.3, p = 0.10 **-0.43**, p = 0.00	**0.98**, p = 0.00 -0.08, p = 0.56 **2.9**, p = 0.002	-0.07, p = 0.34 0.62, p = 0.33 4.6, p = 0.08	**0.50**, p = 0.01 1.1, p = 0.67 -1.5, p = 0.09
**rs12342331**	ASTN2 | TLR4	uLR pMLE-DX pMLE-DX*	**-0.25**, p = 0.00 -1.5, p = 0.13 **-0.25**, p = 0.00	**-0.43**, p = 0.00 -2.5, p = 0.11 **-0.43**, p = 0.00	0.75, p = 0.08 -0.80, p = 0.50 **1.4**, p = 0.01	0.07, p = 0.59 -0.59, p = 0.38 **5.0**, p = 0.03	0.68, p = 0.08 1.8, p = 0.29 -0.35, p = 0.09
**rs16905754**	ASTN2 | TLR4	uLR pMLE-DX pMLE-DX*	**-0.24**, p = 0.00 -1.5, p = 0.05 **-0.24**, p = 0.00	**-0.43**, p = 0.00 -2.7, p = 0.06 **-0.43**, p = 0.00	**1.1**, p = 0.01 5.0, p = 0.07 0.11?, p = 0.00	0.52, p = 0.33 -0.99, p = 0.41 5.0, p = 0.66	**-1.1**, p = 0.002 -4.1, p = 0.21 0.19, p = 0.08
**rs2771054**	ASTN2 | TLR4	uLR pMLE-DX pMLE-DX*	**-0.24**, p = 0.00 -1.5, p = 0.22 **-0.24**, p = 0.00	**-0.43**, p = 0.00 -2.4, p = 0.20 **-0.43**, p = 0.00	**2.9**, p = 0.01 **5.0**, p = 0.04 **-0.19**, p = 0.10	**1.5**, p = 0.01 **4.9**, p = 0.04 **5.0**, p = 0.15	**-1.5**, p = 0.016 -4.1, p = 0.19 1.7, p = 0.66
**rs521581**	ASTN2 | TLR4	uLR pMLE-DX pMLE-DX*	**-0.25**, p = 0.002 -1.7, p = 0.11 **-0.25**, p = 0.00	**-0.43**, p = 0.00 -3.0, p = 0.08 **-0.43**, p = 0.00	**1.1**, p = 0.00 0.35, p = 0.58 1.5, p = 0.10	-0.02, p = 0.43 0.82,p = 0.33 3.5, p = 0.62	**0.34**, p = 0.04 0.61, p = 0.38 -0.36, p = 0.32
**rs1329063**	ASTN2 | TLR4	uLR pMLE-DX pMLE-DX*	**-0.24**, p = 0.002 -1.5, p = 0.19 **-0.24**, p = 0.00	**-0.43**, p = 0.00 -2.8, p = 0.15 **-0.43**, p = 0.00	**1.6**, p = 0.00 0.92, p = 0.34 -2.0, p = 0.17	**0.65**, p = 0.02 -0.10, p = 0.54 5.0, p = 0.58	-0.23, p = 0.33 -0.01, p = 0.57 3.3, p = 0.09
**rs495083**	ASTN2 | TLR4	uLR pMLE-DX pMLE-DX*	**-0.25**, p = 0.00 -1.5, p = 0.11 **-0.24**, p = 0.00	**-0.42**, p = 0.00 -3.3, p = 0.08 **-0.42**, p = 0.00	**1.2**, p = 0.01 1.5, p = 0.25 **4.7**, p = 0.00	-0.19, p = 0.10 0.37, p = 0.28 4.6, p = 0.10	0.24, p = 0.10 -0.72, p = 0.33 **-2.8**, p = 0.04
**rs476**	ASTN2 | TLR4	uLR pMLE-DX pMLE-DX*	**-0.24**, p = 0.00 -1.5, p = 0.13 **-0.24**, p = 0.00	**-0.43**, p = 0.00 -3.0, p = 0.07 **-0.43**, p = 0.00	**3.0**, p = 0.00 2.3, p = 0.20 0.85, p = 0.12	**4.9**, p = 0.03 1.1, p = 0.19 3.2, p = 0.36	-1.3, p = 0.11 -1.6, p = 0.29 0.50, p = 0.64
**rs565055**	ASTN2 | TLR4	uLR pMLE-DX pMLE-DX*	**-0.25**, p = 0.00 -1.3, p = 0.16 **-0.25**, p = 0.002	**-0.43**, p = 0.00 -2.1, p = 0.11 **-0.42**, p = 0.00	**1.1**, p = 0.00 0.81, p = 0.67 1.0, p = 0.21	-0.01, p = 0.47 0.15, p = 0.65 0.84, p = 0.59	**0.37**, p = 0.01 0.14,p = 0.57 2.1, p = 0.33
**rs2094630**	ASTN2 | TLR4	uLR pMLE-DX pMLE-DX*	**-0.25**, p = 0.00 -1.3, p = 0.15 **-0.25**, p = 0.00	**-0.43**, p = 0.00 -2.1, p = 0.11 **-0.43**, p = 0.00	**1.1**, p = 0.00 0.76, p = 0.33 0.30, p = 0.05	-0.2, p = 0.00 0.09, p = 0.57 1.7, p = 0.44	**0.38**, p = 0.02 0.20, p = 0.57 0.32, p = 0.35
**rs10983712**	ASTN2 | TLR4	uLR pMLE-DX pMLE-DX*	**-0.24**, p = 0.00 -1.4, p = 0.16 **-0.25**, p = 0.00	**-0.43**, p = 0.00 **-4.8**, p = 0.03 **-0.43**, p = 0.00	**1.4**, p = 0.00 1.8, p = 0.27 1.3, p = 0.10	0.02, p = 0.59 0.09, p = 0.62 3.0, p = 0.36	-0.04, p = 0.44 -1.0, p = 0.39 0.009, p = 0.49
**rs10983736**	ASTN2 | TLR4	uLR pMLE-DX pMLE-DX*	**-0.24**, p = 0.00 -1.5, p = 0.14 **-0.25**, p = 0.00	**-0.43**, p = 0.00 -2.8, p = 0.09 **-0.43**, p = 0.00	**1.1**, p = 0.01 -0.00, p = 0.67 **2.8**, p = 0.00	-0.24, p = 0.27 -0.67, p = 0.34 **5.0**, p = 0.038	0.26, p = 0.26 0.95, p = 0.65 0.05, p = 0.50
**rs16905962**	ASTN2 | TLR4	uLR pMLE-DX pMLE-DX*	**-0.24**, p = 0.00 -1.4, p = 0.23 **-0.24**, p = 0.00	**-0.43**, p = 0.00 -2.5, p = 0.15 **-0.43**, p = 0.00	-0.005, p = 0.56 -0.51, p = 0.51 -1.5, p = 0.06	-0.14, p = 0.48 **5.1**, p = 0.03 5.0, p = 0.57	1.4,p = 0.15 1.4, p = 0.72 3.1, p = 0.49
**Rs1927914**	ASTN2 | TLR4	uLR pMLE-DX pMLE-DX*	**-0.24**, p = 0.00 -1.3, p = 0.15 **-0.25**, p = 0.00	**-0.43**, p = 0.00 -2.1, p = 0.14 **-0.43**, p = 0.00	**1.4**, p = 0.00 1.2, p = 0.25 1.2, p = 0.12	0.17, p = 0.12 0.65, p = 0.76 3, p = 0.33	0.04, p = 0.58 -0.29, p = 0.47 1.2, p = 0.08
**rs11536879**	TLR4	uLR pMLE-DX pMLE-DX*	**-0.25**, p = 0.00 -1.5, p = 0.07 **-0.24**, p = 0.00	**-0.43**, p = 0.00 -3, p = 0.056 **-0.43**, p = 0.00	0.63, p = 0.25 -2.9, p = 0.37 **-0.28**, p = 0.04	-0.56, p = 0.30 -3.6, p = 0.36 5, p = 0.19	0.78, p = 0.27 3.8, p = 0.25 0.85, p = 0.19
**rs4986790**	TLR4	uLR pMLE-DX pMLE-DX*	**-0.24**, p = 0.00 -1.4, p = 0.08 **-0.24**, p = 0.00	**-0.43**, p = 0.00 -2.4, p = 0.06 **-0.43**, p = 0.00	**3.5**, p = 0.02 **4.9**, p = 0.01 **0.84**, p = 0.00	0.14, p = 0.36 -0.55, p = 0.48 5.0, p = 0.08	**-3.3**, p = 0.02 -4.1, p = 0.20 -1.3, p = 0.26
**rs7045953**	TLR4 | LOC100129489	uLR pMLE-DX pMLE-DX*	**-0.24**, p = 0.00 -1.4, p = 0.10 **-0.24**, p = 0.00	**-0.43**, p = 0.00 -2.4, p = 0.09 **-0.43**, p = 0.00	**0.96**, p = 0.01 -0.19, p = 0.54 **2.6**, p = 0.00	-0.02, p = 0.46 1.6, p = 0.19 4.8, p = 0.47	0.47, p = 0.11 1.1, p = 0.67 0.06, p = 0.44
**rs7357627**	TLR4 | LOC100129489	uLR pMLE-DX pMLE-DX*	**-0.24**, p = 0.00 -1.7, p = 0.11 **-0.24**, p = 0.00	**-0.43**, p = 0.00 -3.3, p = 0.10 **-0.41**, p = 0.00	**1.3**, p = 0.00 1.1, p = 0.28 0.51, p = 0.67	-0.02, p = 0.43 -0.31, p = 0.38 2.3, p = 0.09	0.07, p = 0.63 -0.24, p = 0.43 1.7, p = 0.08
**rs7046020**	TLR4 | LOC100129489	uLR pMLE-DX pMLE-DX*	**-0.24**, p = 0.00 -1.7, p = 0.14 **-0.25**, p = 0.00	**-0.43**, p = 0.00 -2.8, p = 0.11 **-0.42**, p = 0.00	**1.5**, p = 0.00 1.7, p = 0.75 **-4.2**, p = 0.00	0.08, p = 0.69 1.1, p = 0.24 4.5, p = 0.10	-0.11, p = 0.33 -0.91, 0.36 **4.6**, p = 0.01
**rs1927937**	TLR4 | LOC100129489	uLR pMLE-DX pMLE-DX*	**-0.24**, p = 0.00 -1.5, p = 0.11 -0.25, p = 0.00	**-0.43**, p = 0.00 -2.7, p = 0.09 -0.42, p = 0.00	**1.3**, p = 0.00 0.85, p = 0.59 1.3, p = 0.07	-0.02, p = 0.45 0.25, p = 0.66 4.3, p = 0.18	0.08, p = 0.63 0.05, p = 0.50 -0.51, p = 0.20
**rs1927924**	TLR4 | LOC100129489	uLR pMLE-DX pMLE-DX*	**-0.24**, p = 0.00 -1.5, p = 0.23 **-0.24**, p = 0.00	**-0.43**, p = 0.00 -2.9, p = 0.21 **-0.43**, p = 0.00	**1.6**, p = 0.00 **4.9**, p = 0.001 0.15, p = 0.61	0.25, p = 0.18 **4.3**, p = 0.05 5.0, p = 0.25	-0.20, p = 0.28 -4.2, p = 0.17 **1.6**, p = 0.04
**rs3860141**	TLR4 | LOC100129489	uLR pMLE-DX pMLE-DX*	**-0.24**, p = 0.00 -1.2, p = 0.16 **-0.24**, p = 0.00	**-0.42**, p = 0.00 -2.2, p = 0.11 **-0.42**, p = 0.00	**1.1**, p = 0.00 0.01, p = 0.55 1.4, p = 0.17	-0.06, p = 0.33 -0.35, p = 0.35 3.6, p = 0.53	**0.37**, p = 0.02 1.1, p = 0.69 -0.57, p = 0.46
**rs1877876**	TLR4 | LOC100129489	uLR pMLE-DX pMLE-DX*	**-0.24**, p = 0.00 -1.5, p = 0.10 **-0.24**, p = 0.00	**-0.43**, p = 0.00 -2.3, p = 0.08 **-0.42**, p = 0.00	**1.7**, p = 0.00 2.3, p = 0.30 -1.2, p = 0.07	0.20, p = 0.11 1.1, p = 0.22 3.9, p = 0.56	-0.31, p = 0.09 -1.5, p = 0.32 **2.3**, p = 0.02
**rs497322**	TLR4 | LOC100129489	uLR pMLE-DX pMLE-DX*	**-0.25**, p = 0.00 -1.4, p = 0.23 **-0.24**, p = 0.00	**-0.43**, p = 0.00 -1.9, p = 0.20 **-0.43**, p = 0.00	**1.7**, p = 0.01 1.8, p = 0.26 **0.24**, p = 0.03	**0.69**, p = 0.01 **4.5**, p = 0.04 **5.0**, p = 0.05	-0.28, p = 0.28 -1, p = 0.42 1.7, p = 0.47
**rs6478330**	TLR4 | LOC100129489	uLR pMLE-DX pMLE-DX*	**-0.24**, p = 0.00 -1.5, p = 0.15 **-0.25**, p = 0.00	**-0.43**, p = 0.00 -2.6, p = 0.13 **-0.43**, p = 0.00	**1.2**, p = 0.03 -0.008, p = 0.62 **-2.8**, p = 0.048	0.36, p = 0.21 0.73, p = 0.24 5.0, p = 0.14	0.16, p = 0.28 0.93, p = 0.33 **5.5**, p = 0.01
**rs7856175**	TLR4 | LOC100129489	uLR pMLE-DX pMLE-DX*	**-0.24**, p = 0.00 -1.4, p = 0.13 **-0.25**, p = 0.00	**-0.43**, p = 0.00 -2.3, p = 0.11 **-0.43**, p = 0.00	**1.7**, p = 0.00 1.6, p = 0.24 **1.5**, p = 0.03	0.07, p = 0.30 0.43, p = 0.28 2.7, p = 0.61	**-0.33**, p = 0.03 -0.97, p = 0.36 -0.08, p = 0.28
**rs3134940**	AGER	uLR pMLE-DX pMLE-DX*	**-0.97,** p = 0.00 -0.74, p = 0.13 -0.96, p = 0.11	-0.12, p = 0.08 -0.52, p = 0.14 -1.2, p = 0.10	**1.1**, p = 0.00 1.2, p = 0.40 1.4, p = 0.09	**0.55**, p = 0.00 1.2, p = 0.18 1.8, p = 0.13	-0.28, p = 0.05 -0.67, p = 0.33 -0.32, p = 0.50
**rs1035798**	AGER	uLR pMLE-DX pMLE-DX*	**-0.97**, p = 0.00 -1.42, p = 0.09 -0.97, p = 0.11	**-0.13**, p = 0.06 -0.29, p = 0.18 -0.60, p = 0.15	**0.89**, p = 0.00 0.96, p = 0.56 1.3, p = 0.08	**0.43**, p = 0.00 0.67, p = 0.21 1.8, p = 0.13	0.03, p = 0.85 -0.06, p = 0.41 -1.9, p = 0.08
**rs2070600**	AGER	uLR pMLE-DX pMLE-DX*	**-0.97**, p = 0.00 -2.3, p = 0.08 -0.97, p = 0.11	-0.12, p = 0.09 -0.50, p = 0.18 -0.62, p = 0.13	**0.99**, p = 0.00 0.97, p = 0.59 1.5, p = 0.05	**0.49**, p = 0.00 0.99, p = 0.22 1.8, p = 0.13	**-0.14**, p = 0.33 -0.24, p = 0.17 -0.23, p = 0.51

Analyses are performed using the usual logistic regression (uLR) that uses the clinical diagnosis as an outcome and using pseudo-likelihood method that assumes that the proportion of nuisance disease within the clinically diagnosed AD is 36% for *ε*4 non-carriers and is 6% for *ε*4 carriers. Pseudo-likelihood analyses pMLE-DX estimates parameters for *D* = 1 vs. *D* = 0 and *D* = 1* combined. Pseudo-likelihood analyses pMLE – DX*, however, estimate two sets of risk coefficients, i.e. *β*s for *D* = 0 vs. *D* = 1 and β*s D = 0 vs. *D* = 1*. Estimates of β*s are reported in **S7 Table**.

Estimates of *β*_*G*_ or *β*_*G*×*ε*4_ differ numerically between the three approaches. For 14 of these 53 SNPs, ${\hat{\beta }}_{G\times \varepsilon 4}$ have p-values <0.05 in uLR, while in pMLE-DX and pMLE-DX* the corresponding p-values are >0.05. These associations detected by uLR might be spurious as a result of clinical-pathophysiological diagnoses relationship varying by ApoE *ε*4 status.

One SNP, rs830832, has significant ${\hat{\beta }}_{G\times \varepsilon 4}$ both in uLR (${\hat{\beta }}_{G\times \varepsilon 4}=0.74,p=0.01$) and ${\mathrm{pMLE}‑\mathrm{DX}}^{*}({\hat{\beta }}_{\left(G\times \varepsilon 4\right)}=2.6,p=0.03)$. This SNP locates at the intergenic region between SORBS2 and TLR3 at Chromosome 4 and are 72k downstream of SNP rs75718659, which was reported associated with Alzheimer’s disease in a family-based GWAS [10].

Among the seven SNPs appear to have significant ${\hat{\beta }}_{G\times \varepsilon 4}$ in pMLE- DX* but not uLR, two of the SNPs: rs4862611 (${\hat{\beta }}_{G\times \varepsilon 4}$ = -2.8, p = 0.03) and rs1706143 (${\hat{\beta }}_{G\times \varepsilon 4}$ = 2.9, p = 0.03), are also located at the intergenic region between SORBS2 and TLR3 at Chromosome 4 and are 80k and 20k downstream of SNP rs75718659.

Nine of the SNPs appear to have significant ${\hat{\beta }}_{G\times \varepsilon 4}^{*}$ in pMLE-DX* but not ${\hat{\beta }}_{G\times \varepsilon 4}$ in uLR or pMLE-DX. Two SNPs, rs7676342 (${\hat{\beta }}_{G\times \varepsilon 4}^{*}=-2.1,p=0.02$) and rs13113778 (${\hat{\beta }}_{G\times \varepsilon 4}^{*}=-2.7,p=0.03$), again are located in the intergenic region between SORBS2 and TLR3 at Chromosome 4 and are 80k and 100k downstream of SNP rs75718659, respectively. Three SNPs, rs955302 (${\hat{\beta }}_{G\times \varepsilon 4}^{*}=4.0,p=0.01$), rs4837254 (${\hat{\beta }}_{G\times \varepsilon 4}^{*}=2.3,p=0.04$) and rs12342331 (${\hat{\beta }}_{G\times \varepsilon 4}^{*}=2.1,p=0.04$), are located at the intergenic region between ASTN2 and TLR4 at Chromosome 9 and are 400k, 430k, and 492k downstream of rs1360695 associated with Schizophrenia [11].

Estimates of *β*_*G*_, however, are generally larger in magnitude when estimated in pMLE-DX and pMLE- DX* models.

Two SNPs appear to be associated with the diagnosis both in uLR and pMLE-DX. SNP rs7656500 (uLR ${\hat{\beta }}_{G}=0.79,p=0.01$ and pMLE-DX ${\hat{\beta }}_{G}=6,p=0.02$) locates at the intergenic region between KIAA0922 and TLR2 at Chromosome 4, and is 163k upstream and 144k downstream of rs727153 and rs1466662, respectively, which were reported associated with Alzheimer’s disease in two studies [12,13]. It is also 54k upstream of rs7654093 associated with thrombosis [14], 30k upstream of rs7659024 associated with Venous thromboembolism [15], 34k upstream of rs2066865 associated with Venous thromboembolism [16, 17], 52k upstream of rs6536024 associated with Venous thromboembolism [18], and 360k downstream of rs11099942 associated with Type 2 diabetes [19].

Among six SNPs which appear to be significantly associated with the nuisance diagnosis in absence of an interactive effect, three SNPs rs1869617 (at the intergenic region between SORBS2 and TLR3 at Chromosome 4, pMLE- $DX^{*}\;{\hat{\beta }}_{G}^{*}=4.9,p=0.01$), rs3775296 (at the UTR region of TLR3 at Chromosome 4, pMLE- $DX^{*}\;{\hat{\beta }}_{G}^{*}=4.3,p=0.046$), rs7668666 (at the INTRON region of TLR3 at Chromosome 4, pMLE- $DX^{*}\;{\hat{\beta }}_{G}^{*}=4.9,p=0.00$) locate 110k, 176k and 179k, respectively, downstream of rs75718659 reported associated with Alzheimer’s disease [10] and another two SNPs rs16905625 (pMLE- $DX^{*}\;{\hat{\beta }}_{G}^{*}=3.2,p=0.03$) and rs1890047 (pMLE- ${\mathrm{DX}}^{*}\;{\hat{\beta }}_{{}_{G}}^{*}=4.6,p=0.008$) locate at the intergenic region between ASTN2 and TLR4 at Chromosome 9, 412k and 428k, respectively downstream of rs1360695 reported associated with Schizophrenia [11].

Estimates of *β*_*ε*4_ in the absence of interaction are generally larger in magnitude for the diagnosis of interest in pMLE-DX. For example, in a model with SNP rs1816702 (uLR ${\hat{\beta }}_{\varepsilon 4}=1.4,p=0.00$ and pMLE- $DX^{*}\;{\hat{\beta }}_{\varepsilon 4}=2.6,p=0.03,{\hat{\beta }}_{\varepsilon 4}^{*}=1.2,p=0.01$).

#### AGER

All of the three SNPs in the AGER gene measured in the data are associated with susceptibility to AD as inferred in uLR and also are associated with susceptibility to the nuisance disease when measured by pMLE- DX*. **rs3134940** has been previously reported in association to breast cancer, type I diabetes and other phenotypes (https://www.gwascentral.org/marker/HGVM1600838/results?t=ZERO); **rs1035798** and **rs2070600** have been previously reported in association to rheumatoid arthritis (https://www.gwascentral.org/marker/HGVM275161/results?t=ZERO and https://www.gwascentral.org/marker/HGVM571318/results?t=ZERO).

## Discussion

We investigated if disease heterogeneity among clinically diagnosed cases could introduce bias into the estimates of GxE interactions. We showed that when there is a strong association between the environmental variable and the relative risk of the disease of interest, as compared to the nuisance disease, and then there could be bias in either direction. We base our developments on the method by Chatterjee and Carroll [7] that is fully efficient in situations when the genetic and environmental variables are distributed independently in the population, a population-based genetics model is assumed for the genetic factors and the environmental variables are treated non-parametrically.

Interestingly, in our analyses, the estimates of regression coefficients are qualitatively differed between the analyses that used the clinical diagnosis as a surrogate of the pathologic diagnosis and the analyses that used our newly proposed pseudo-likelihood approach that incorporates the uncertainty of the clinical diagnosis. Specifically, in TLR set for 13% of the SNPs examined, GxE was found to be significant in the relationship to the clinical diagnosis, while the pseudo-likelihood analyses inferred these GxE to be not significant. On the other hand, for 14% of the SNPs that we examined, GxE was found to be statistically significant only when we incorporated the uncertainty in the clinical-pathological diagnoses relationship. This finding is consistent with the conclusion reached by a study of phenotypic misclassification among cases [20] in situations when the misclassification is non-differential, i.e. is not a function of the environmental variables. The study concluded that presence of “non-cases” greatly decreased the estimates of risk attributed to the genetic variation.

One of the major concerns in the analyses of the genetic studies has been the missing heritability, when the genetic markers identified thus far explain only a small portion of inter-person variability in familiar clustering of complex diseases [21]. The downward biases in the estimates associating GxE to the clinically diagnosed disease status might in part explain the missing heritability. On the other hand, the upward biases in these estimates might in part address the conclusion reached by [22] that only 1% of the association found are likely to be true.

We examined estimates of the genetic effects, ApoE4 status, and age, sex consistent with the original publication on this dataset [23]. Epidemiologic evidence [24] suggests that the following factors play important role in AD risk: education/cognitive reserve, racial and ethnic difference, gender, smoking, drinking, head injury, diabetes, cardiovascular disease, obesity, social engagement, etc. However, not all of these factors have been consistently confirmed by subsequent studies, and considerable inconsistencies exist. For example, nicotine intake has been observed to decrease the risk of dementia due to the demonstrated ability of nicotine to stimulate neurotransmitter systems that are compromised in dementia [25]. More recent studies have suggested that nicotine intake may increase the risk of AD and also bring forward age of onset with APOE interactive effect [26].

The main conclusion reached in this paper is that using the clinically diagnosed status can lead to severely biased estimates of GxE interactions in situations when the frequency of the pathologic diagnosis of interest, as compared to other diagnoses, depends on the environment, and we aim to correct such biases by proposing pseudolikelihood method. AD dataset is mainly used for illustration, therefore, for clarity we restricted to variables to the minimum necessary instead of considering full risk prediction modes which might be able to better describe the inter-patient variability in susceptibility to AD. Although other factors are potentially important in predicting the risk of AD, this relatively simple model was able to achieve the main goals of the current manuscript. By recognizing and accounting for the potential of case heterogeneity, which biases the gene x environment interaction, our newly proposed method has the ability to remove this bias.

Define *E* to be the set of variables in the model, i.e. age, sex. Let *O* define a set of key environmental variables omitted from the model. Addition of variables *O* would not modify the effect estimates of GxE beyond what is expected purely by chance if *O* does not interact with either *G* or *E*. Also, if conditional on the diagnosis of AD, GxE is independent of *O*, then omission of *O* does not change the effect estimate of GxE [27]. If, however, *O* interacts with GxE, then addition of these variables would change the effect estimate of GxE in the direction that is consistent with the direction of the GxE effect. Further studies that incorporate environmental variables, such as medical history, tobacco use, and infections are needed for their potential to modify the risk and the estimates of GxE in particular.

Epigenetic mechanisms are well-recognized in the mediation of GxE and analysis of epigenetic changes at the genome scale can offer new insights into the relationship between brain epigenomes and AD. Further, candidate genes from epigenome-wide association studies interact with those from GWAS that can undergo epigenetic changes in their upstream gene regulatory elements [28]. However, an active conundrum is how the epigenetic mechanisms influence gene-environment interactions.

## Appendix

### Derivation of pseudo-likelihood (2) and covariance matrix

Derivation of the pseudo-likelihood (2) is straightforward.

Next we demonstrate that the pseudo-likelihood (2) has zero mean when evaluated at the true parameters. Derivative of (2) with respect to Ω is

$$\frac{\sum_{d^{∎}}\gamma_{d^{∎}|d^{cl}}\left(x\right)\times S_{\Omega }\left(d^{∎},d^{cl},g,x,z;\Omega \right)}{\sum_{d^{∎}}\gamma_{d^{∎}|d^{cl}}\left(x\right)\times S\left(d^{∎},d^{cl},g,x,z;\Omega \right)}-\frac{\sum_{d^{∎},g^{∎},d^{cl∎}}\gamma_{d^{∎}|d^{cl∎}}\left(x\right)\times S_{\Omega }\left(d^{∎},d^{cl},g^{∎},x,z;\Omega \right)}{\sum_{d^{∎},g^{∎},d^{cl∎}}\gamma_{d^{∎}|d^{cl∎}}\left(x\right)\times S\left(d^{∎},d^{cl},g^{∎},x,z;\Omega \right)}=A\left(d^{cl},g,x,z\right)-B\left(x,z\right).$$

Let p(*x*,*z*|*η*) be the density of the environment.

Note the conditional probabilities

$$[G,X,Z|D^{CL}]=n_{d^{cl}}^{-1}\sum_{d^{∎}}{\gamma_{d^{cl}|d^{∎}}(x)\times S(d^{∎},d^{cl},g,x,z;\Omega ),}_{}$$

$$[X,Z|D^{CL}]=n_{d^{cl}}^{-1}\sum_{{g^{∎},d}^{∎}}\gamma_{d^{cl}|d^{∎}}(x)\times S(d^{∎},d^{cl},g^{∎},x,z;\Omega )\times p(x,z|\eta ).$$

Hence

$$E\{A(D^{CL},G,X,Z)\}=\sum_{d^{c*}}\frac{n_{d^{cl∎}}}{n}E\{A(D^{CL},G,X,Z)|D^{CL}=d^{cl∎}\}$$

$$=\frac{1}{n}\sum_{{d^{∎},d}^{cl∎},g^{∎},x^{∎},z^{∎}}\gamma_{d^{cl}|d^{∎}}(x^{∎})\times S_{\Omega }(d^{∎},d^{cl∎},g^{∎},x^{∎},z^{∎};\Omega )\times p(x^{∎},z^{∎}|\eta )$$

$$=\sum_{d^{cl∎}}\frac{n_{d^{cl∎}}}{n}E\{B(X,Z)|D^{CL}=d^{cl∎}\}=E\{B(X,Z)\}.$$

Therefore the derivative of the pseudo-likelihood has zero mean when evaluated at the true parameters. Evaluated at the true parameters the estimating function (2) takes the following form

$$n^{-1/2}\sum_{i=1}^{n}E[A(d^{cl},g,x,z)-B(x,z)-E\{A(d^{cl},g,x,z)-B(x,z)|D^{CL}=d^{cl}\}].$$

Covariance matrix is then

$$\Sigma =n^{-1}\sum_{i=1}^{n}E[\{A(d^{cl},g,x,z)-B(x,z)\}\times \{A(d^{cl},g,x,z)-B(x,z){\}}^{T}]-\Lambda .$$

Define

$$Q_{1}(d^{cl},g,x,z;\Omega )=\sum_{d^{∎}}\gamma_{d^{cl}|d^{∎}}(x)\times S_{\Omega }(d^{∎},d^{cl},g,x,z;\Omega )\times p(x,z|\eta );$$

$$Q_{2}(d^{cl},g,x,z;\Omega )=\sum_{d^{∎}}\gamma_{d^{cl}|d^{∎}}(x)\times S(d^{∎},d^{cl},g,x,z;\Omega )\times p(x,z|\eta );$$

$$Q_{3}(x,z;\Omega )=\sum_{d^{cl∎},d^{∎},g^{∎}}\gamma_{d^{cl}|d^{∎}}(x)\times S_{\Omega }(d^{∎},d^{cl∎},g^{∎},x,z;\Omega )\times p(x,z|\eta );$$

$$Q_{4}(x,z;\Omega )=\sum_{d^{cl∎},d^{∎},g^{∎}}\gamma_{d^{cl}|d^{∎}}(x)\times S(d^{∎},d^{cl∎},g^{∎},x,z;\Omega )\times p(x,z|\eta );$$

$$\Sigma_{1}=n^{-1}\sum_{d^{cl∎},g^{∎},x^{∎},z^{∎}}\frac{Q_{1}(d^{cl∎},g^{∎},x^{∎},z^{∎};\Omega )\times Q_{1}^{T}(d^{cl∎},g^{∎},x^{∎},z^{∎};\Omega )}{Q_{2}(d^{cl∎},g^{∎},x^{∎},z^{∎};\Omega )}p(x^{∎},z^{∎}|\eta );$$

$$\Sigma_{2}=n^{-1}\sum_{x^{∎},z^{∎}}\frac{Q_{3}(x^{∎},z^{∎};\Omega )\times Q_{3}^{T}(x^{∎},z^{∎};\Omega )}{Q_{4}(x^{∎},z^{∎};\Omega )}p(x^{∎},z^{∎}|\eta ).$$

The covariance matrix can then be represented in the form Σ = Σ_1_-Σ_2_−Λ.

Define $I_{1}=\sum_{d^{cl∎}}\frac{n_{d^{cl∎}}}{n}E[\frac{\partial }{\partial \Omega }\{\frac{Q_{1}(d^{cl∎},g,x,z;\Omega )}{Q_{2}(d^{cl∎},g,x,z;\Omega )}|D^{CL}=d^{cl}\}]$ and $I_{2}=\sum_{d^{cl∎}}\frac{n_{d^{cl∎}}}{n}E[\frac{\partial }{\partial \Omega }\{\frac{Q_{3}(d^{cl∎},g,x,z;\Omega )}{Q_{4}(d^{cl∎},g,x,z;\Omega )}|D^{CL}=d^{cl}\}]$, then *I* = *I*_1_−*I*_2_.

We note that $I_{1}=n^{-1}\frac{\partial^{2}}{\partial \Omega \partial \Omega^{T}}\sum_{d^{cl∎},d^{∎},g^{∎},x^{∎},z^{∎}}\gamma_{d^{cl}|d^{∎}}(x^{∎})\times S_{\Omega }(d^{∎},d^{cl∎},g^{∎},x^{∎},z^{∎};\Omega )\times p(x^{∎},z^{∎}|\eta )+\Sigma_{1}$ and $I_{2}=n^{-1}\frac{\partial^{2}}{\partial \Omega \partial \Omega^{T}}\sum_{d^{cl∎},d^{∎},g^{∎},x^{∎},z^{∎}}\gamma_{d^{cl}|d^{∎}}(x^{∎})\times S_{\Omega }(d^{∎},d^{cl∎},g^{∎},x^{∎},z^{∎};\Omega )\times p(x^{∎},z^{∎}|\eta )+\Sigma_{2}$. Hence Σ = *I*_1_−*I*_2_−Λ = *I*−Λ.

## Supporting information

S1 Table *β*_*G×ε*__4_ ≠ 0. The Bias and Root Mean Squared Error (RMSE) in parameter estimates from simulations using the usual logistic regression with clinical diagnosis as the outcome (uLR), the pseudo-likelihood approach (pMLE), and our newly proposed pseudo-likelihood approach that accounts for misdiagnosis (pMLE-DX). For these simulations, the study included *n*_0_ controls and *n*_1_ cases. Frequency of ApoE *ε*4 allele in the population is 14%. Variables *Z*_1_ and *Z*_2_ are Bernoulli with frequencies 0.50 and 0.52, respectively. Frequency of the *true* disease status is 46% in the population; and is 40% among the subpopulation with no ApoE *ε*4 alleles, and 82% in the subpopulation with at least one ApoE *ε*4 alleles. Frequency of nuisance disease within the clinical diagnosis varies by ApoE4 status pr(D = 1*|*D*^*CL*^ = 1,*ε*4−) = 0.36 and pr(D = 1*|*D*^*CL*^ = 1,*ε*4) = 0.06.
(DOCX)

S2 Table *β*_*G*×*ε*4_ = 0. The Bias and Root Mean Squared Error (RMSE) in parameter estimates from simulations using the usual logistic regression with clinical diagnosis as the outcome (uLR), the pseudo-likelihood approach (pMLE), and our newly proposed pseudo-likelihood approach that accounts for misdiagnosis (pMLE-DX). For these simulations, the study included *n*_0_ controls and *n*_1_ cases. Frequency of ApoE *ε*4 allele in the population is 14%. Variables *Z*_1_ and *Z*_2_ are Bernoulli with frequencies 0.50 and 0.52, respectively. Frequency of the *true* disease status is 46% in the population; and is 40% among the subpopulation with no ApoE *ε*4 alleles, and 82% in the subpopulation with at least one ApoE *ε*4 alleles. Frequency of nuisance disease within the clinical diagnosis varies by ApoE4 status pr(D = 1*|*D*^*CL*^ = 1,*ε*4−) = 0.36 and pr(D = 1*|*D*^*CL*^ = 1,*ε*4+) = 0.06.
(DOCX)

S3 Table Frequency of the nuisance disease is underestimated. The Bias and Root Mean Squared Error (RMSE) in parameter estimates from simulations using the usual logistic regression with clinical diagnosis as the outcome (uLR), the pseudo-likelihood approach (pMLE), and our newly proposed pseudo-likelihood approach that accounts for misdiagnosis (pMLE-DX). For these simulations, the study included *n*_0_ = 3000 controls and *n*_1_ = 3000 cases. Frequency of ApoE *ε*4 allele in the population is 14%. Variables *Z*_1_ and *Z*_2_ are Bernoulli with frequencies 0.50 and 0.52, respectively. Frequency of the *true* disease status is 46% in the population; and is 40% among the subpopulation with no ApoE *ε*4 alleles, and 82% in the subpopulation with at least one ApoE *ε*4 alleles. Frequency of nuisance disease within the clinical diagnosis varies by ApoE4 status pr(D = 1*|*D*^*CL*^ = 1,*ε*4−) = 0.36 and pr(D = 1*|*D*^*CL*^ = 1,*ε*4+) = 0.06. The clinical-pathophysiological diagnoses relationship is misspecified to be pr(D = 1*|*D*^*CL*^ = 1,*ε*4−) = 0.30 and pr(D = 1*|*D*^*CL*^ = 1,*ε*4+) = 0.
(DOCX)

S4 Table Frequency of the nuisance disease is overestimated. Bias and Root Mean Squared Error (RMSE) for parameter estimates based on a study of 500 simulated datasets with *n*_0_ controls and *n*_1_ cases with clinical phenotype. Analyses are based on the usual logistic regression model that ignores nuisance disease and based on pseudolikelihood with (pMLE-DX) and without the consideration of clinical-pathological diagnoses relationship (pMLE). Frequency of ApoE *ε*4 alleles is 14% in the population. Variables *Z*_1_ and *Z*_2_ are Bernoulli with frequencies 0.50 and 0.52, respectively. Frequency of the *true* disease status is 46% in the population; and is 40% among the subpopulation with no ApoE *ε*4 alleles, and 82% in the subpopulation with at least one ApoE *ε*4 alleles. Frequency of nuisance disease within the clinical diagnosis varies by ApoE4 status pr(D = 1′|*D*^*CL*^ = 1,*ε*4−) = 0.36 and pr(D = 1′|*D*^*CL*^ = 1,*ε*4+) = 0.06. The clinical-pathological diagnoses relationship is misspecified to be pr(D = 1′|*D*^*CL*^ = 1,*ε*4−) = 0.42 and pr(D = 1′|*D*^*CL*^ = 1,*ε*4+) = 0.12.
(DOCX)

S5 Table$\beta_{G\times \varepsilon 4}^{*}=0$ and $\beta_{G}^{*}=0$.The Bias and Root Mean Squared Error (RMSE) in parameter estimates from simulations using the usual logistic regression with clinical diagnosis as t he outcome (uLR), the pseudo-likelihood approach (pMLE), and our newly proposed pseudo-likelihood approach that accounts for misdiagnosis (pMLE-DX). For these simulations, the study included *n*_0_ controls and *n*_1_ cases. Risk of the disease of interest is defined in a set of parameters $\beta_{0},\beta_{G},\beta_{Z_{1}},\beta_{Z_{2}},\beta_{G\times \varepsilon 4}$; while the risk of the nuisance disease is parametrized by $\beta_{0}^{*},\beta_{G}^{*},\beta_{\varepsilon 4}^{*},\beta_{G\times \varepsilon 4}^{*}.$ Frequency of ApoE *ε*4 allele in the population is 14%. Variables *Z*_1_ and *Z*_2_ are Bernoulli with frequencies 0.50 and 0.52, respectively. Frequencies of the disease of interest and the nuisance disease are pr(D = 1) = 24.8%, pr(D = 1*) = 12.5%, pr(D = 1|*ε*4+) = 43%, pr(D = 1*|*ε*4+) = 16.1%, pr(D = 1|*ε*4−) = 20%, pr(D = 1*|*ε*4−) = 11.6%. Frequency of the nuisance disease within the clinical diagnosis varies by ApoE4 status pr(D = 1*|*D*^*CL*^ = 1,*ε*4−) = 0.36 and pr(D = 1*|*D*^*CL*^ = 1,*ε*4+) = 0.06.(DOCX)

S6 Table*β*_*G*×*ε*4_ = 0, $\beta_{G\times \varepsilon 4}^{*}=0,\beta_{G}^{*}=0$.The Bias and Root Mean Squared Error (RMSE) in parameter estimates from simulations using the usual logistic regression with clinical diagnosis as the outcome (uLR), the pseudo-likelihood approach (pMLE), and our newly proposed pseudo-likelihood approach that accounts for misdiagnosis (pMLE-DX). For these simulations, the study included *n*_0_ controls and *n*_1_ cases. Risk of the disease of interest is defined in a set of parameters $\beta_{0},\beta_{G},\beta_{Z_{1}},\beta_{Z_{2}},\beta_{G\times \varepsilon 4}$; while the risk of the nuisance disease is parametrized by $\beta_{0}^{*},\beta_{G}^{*},\beta_{\varepsilon 4}^{*},\beta_{G\times \varepsilon 4}^{*}$. Frequency of ApoE *ε*4 allele in the population is 14%. Variables *Z*_1_ and *Z*_2_ are Bernoulli with frequencies 0.50 and 0.52, respectively. Frequencies of the disease of interest and the nuisance disease are pr(D = 1) = 24.8%, pr(D = 1*) = 12.5%, pr(D = 1|*ε*4+) = 43%, pr(D = 1*|*ApoE*4+) = 16.1%, pr(D = 1|*ε*4−) = 20%, pr(D = 1*|*ε*4−) = 11.6%. Frequency of the nuisance disease within the clinical diagnosis varies by ApoE4 status pr(D = 1*|*D*^*CL*^ = 1,*ε*4−) = 0.36 and pr(D = 1*|*D*^*CL*^ = 1,*ε*4+) = 0.06.(DOCX)

S7 Table Parameter estimates in Alzheimer’s disease study. Analyses are performed using the usual logistic regression (uLR) that uses the clinical diagnosis as an outcome and using pseudo-likelihood method that assumes that the proportion of nuisance disease within the clinically diagnosed AD is 36% for *ε*4 carriers and is 6% for *ε*4 non-carriers. Pseudo-likelihood analyses pMLE-DX estimates parameters for *D* = 1 vs. *D* = 0 and *D* = 1* combined. Pseudo-likelihood analyses pMLE − DX*, however, estimate two sets of risk coefficients, i.e. *β*s for *D* = 0 vs. *D* = 1 and β*s D = 0 vs. *D* = 1*.
(DOCX)

S8 Table SNPs previously reported in GWAS that are within 500k up- or downstream of SNPs that we inferred in Alzheimer’s disease study. (Table 5, SNPs whose effect estimates of *β*_*G*_ and/or *β*_*G*×*ε*4_ are with permutation-based p-value <0.05).
(DOCX)

## References

[1] Potter H, Wisniewski T. Apolipoprotein E: essential catalyst of the Alzheimer amyloid cascade. International Journal of Alzheimer’s Disease. 2012; 10.1155/2012/489428 PMC3403541 22844635

[2] Salloway S, Sperling R. Understanding conflicting neurological findings in patients clinically diagnosed as having Alzheimer Dementia. JAMA Neurology. 2015; 72 (10): 1106–1108. 10.1001/jamaneurol.2015.1804 26302229

[3] Shaw AC, Panda A, Joshi SR, Qian F, Allore HG, Montgomery RR. Dysregulation of human toll-like receptor function in aging. Ageing Research Review. 2011 7;10(3):346–53. 10.1016/j.arr.2010.10.007 21074638 PMC3633557

[4] Ramasamy R, Vannucci SJ, Yan SSD, Herold K, Yan SF, Schmidt AM. Advanced glycation end products and RAGE: a common thread in aging, diabetes, neurodegeneration, and inflammation. Glycobiology. 2005; 15(7): 16R–28R. 10.1093/glycob/cwi053 15764591

[5] Carroll RJ, Ruppert D, Stefanski LA, Crainiceanu. Measurement error in nonlinear models: a modern perspective. 2nd ed Chapman and Hall/CRC; 2006.

[6] Prentice KL, Pyke DA. Logistic disease incidence models and case-control studies, Biometrika.1979; 66(3): 403–411.

[7] Chatterjee N, Carroll RJ. Semiparametric maximum likelihood estimation exploiting gene-environment independence in case-control studies. Biometrika. 2005; 92(2): 399–418.

[8] Hardy GH. Mendelian Proportions in a Mixed Population. Science. 1908; 28(706): 49–50. 10.1126/science.28.706.49 17779291

[9] Benjamini Y., and Hochberg Y. Controlling the false discovery rate: a practical and powerful approach to multiple testing. Journal of the Royal Statistical Society Series. 1995; B 57: 289–300.

[10] Herold C, Hooli BV, Mullin K, Liu T, Roehr JT, Mattheisen M, et al (2016) Family-based association analyses of imputed genotypes reveal genome-wide significant association of alzheimer’s disease with OSBPL6, PTPRG and PDCL3. Molecular Psychiatry. 2016; 21(11):1608–1612. 10.1038/mp.2015.218 26830138 PMC4970971

[11] Goes FS, McGrath J, Avramopoulos D, Wolyniec P, Pirooznia M, Ruczinki I, et al (2015) Genome-wide association study of schizophrenia in Ashkenazi Jews. American Journal of Medical Genetics. 2015; 168(8):649–659. 10.1002/ajmg.b.32349 26198764

[12] Abraham R, Moskvina V, Sims R, Hollingworth P, Morgan A, Georgieva L, et al A genome-wide association study for late-onset Alzheimer’s disease using DNA pooling, BMC Medical Genomics. 2008; 29: 1–44. 10.1186/1755-8794-1-44 PMC2570675 18823527

[13] Kamboh MI, Barmada MM, Demirci FY, Minster RL, Carrasquillo MM, Pankratz VS, et al Genome-wide association analysis of age-at-onset in Alzheimer's disease. Molecular Psychiatry. 2012 12;17(12):1340–6. 10.1038/mp.2011.135 22005931 PMC3262952

[14] Hinds DA, Buil A, Ziemek D, Martinez-Perez A, Malik R, Folkersen L et al Genome-wide association analysis of self-reported events in 6135 individuals and 252 827 controls identifies 8 loci associated with thrombosis. Human Molecular Genetics. 2016; 25(9):1867–1874. 10.1093/hmg/ddw037 26908601 PMC4986325

[15] Germain M, Saut N, Greliche N, Dina C, Lambert JC, Perret C, et al Genetics of venous thrombosis: insights from a new genome wide association study. PLOS One. 2011; 6(9): e25581 10.1371/journal.pone.0025581 21980494 PMC3181335

[16] Germain M, Chasman DI, de Haan H, Tang W, Lindström S, Weng LC, et al Meta-analysis of 65,734 individuals identifies TSPAN15 and SLC44A2 as two susceptibility loci for venous thromboembolism. American Journal of Human Genetics. 2015 4 2;96(4):532–42. 10.1016/j.ajhg.2015.01.019 25772935 PMC4385184

[17] Klarin D, Emdin CA, Natarajan P, Conrad MF, INVENT Consortium, Kathiresan S. Genetic Analysis of Venous Thromboembolism in UK Biobank Identifies the ZFPM2 Locus and Implicates Obesity as a Causal Risk Factor. Circulation Cardiovascular Genetics. 2017 4;10(2). pii: e001643 10.1161/CIRCGENETICS.116.001643 28373160 PMC5395047

[18] Tang W, Teichert M, Chasman DI, Heit JA, Morange PE, Li GA genome-wide association study for venous thromboembolism: the extended Cohorts for Heart and Aging Research in Genomic Epidemiology (CHARGE) Consortium, Genetic Epidemiology Journal 2013 37(5): 512–521 10.1002/gepi.21731 PMC3990406 23650146

[19] Hamet P, Haloui M, Harvey F, Marois-Blanchet FC, Sylvestre MP, Tahir MR, et al PROX1 gene CC genotype as a major determinant of early onset of type 2 diabetes in slavic study participants from Action in Diabetes and Vascular Disease: Preterax and Diamicron MR Controlled Evaluation study, Journal of Hypertension. 2017 5; 35 Suppl 1:S24–S32. 10.1097/HJH.0000000000001241 28060188 PMC5377997

[20] Manchia M, Cullis J, Gustavo T, Rouleau GY, Uher R, Alda M. The impact of phenotypic and genetic heterogeneity on results of genome-wide association studies of complex diseases. PLOS One. 2013; 8(10): e76295 10.1371/journal.pone.0076295 24146854 PMC3795757

[21] Manolio TA, Collins FS, Cox NJ, Goldstein DB, Hindorf LA, Hunter DJ, et al Finding the missing heritability of complex diseases. 2009 10 8;461(7265):747–53. 10.1038/nature08494 19812666 PMC2831613

[22] Hirschhorn JN, Lohmueller K, Byrne E, Hirschhorn K. A comprehensive review of genetic association studies. Genet Med. 2002; 2:45–61. 10.1097/00125817-200203000-00002 11882781

[23] Naj AC, Jun G, Beecham GW, Wang LS, Vardarajan BN, Buros J, et al Common variants at MS4A4/MS4A6E, CD2AP, CD33 and EPHA1 are associated with late-onset Alzheimer’s. Nature Genetics. 2011 5;43(5):436–41. 10.1038/ng.801 21460841 PMC3090745

[24] Richie K, Carriere I, Richi CW, Berr C, Artero S, Ancelin ML. Designing prevention programs to reduce incidence of dementia: prospective cohort study of modifiable risk factors. British Medical Journal. 2010 8 5;341:c3885 10.1136/bmj.c3885 20688841 PMC2917002

[25] Van Duijn CM, Hofman A. Relation between nicotine intake and Alzheimer’s disease. British Medical Journal. 1991; 22:1491–1494. 10.1136/bmj.302.6791.1491 PMC1670208 1855016

[26] Lee, C., Alekseenko, A., Brown, T. (2009) Exploring the future of bioinformatics data sharing and mining with Pygr and Worldbase, Proceedings of the 8th Python in Science Conference (SciPy 2009)

[27] Hauck WW, Neuhaus JM, Kalbfleisch JD, Anderson S (1991) A consequence of omitted covariates when estimating odds ratios. J Clin Epidemiol; 44(1):77–81 1986061 10.1016/0895-4356(91)90203-l

[28] Hoffman A, Sportelli V, Ziller M, Spengler D. Driver or Passenger: Epigenomes in Alzheimer’s disease. Epigenomes. 2017; 1(1) 5; 10.3390/epigenomes1010005

