# Supplementary material for: Case-control studies of gene-environment interactions. When a case might not be the case
Source: PLoS One. 2018 Aug 22;13(8):e0201140. doi: 10.1371/journal.pone.0201140 (PMC6104951; doi:10.1371/journal.pone.0201140)
Supplement: S2 Table — The Bias and Root Mean Squared Error (RMSE) in parameter estimates from simulations using the usual logistic regression with clinical diagnosis as the outcome (uLR), the pseudo-likelihood approach (pMLE), and our newly proposed pseudo-likelihood approach that accounts for misdiagnosis (pMLE-DX). For these simulations, the study included n0 controls and n1 cases. Frequency of ApoE ε4 allele in the population is 14%. Variables Z1 and Z2 are Bernoulli with frequencies 0.50 and 0.52, respectively. Frequency of the true disease status is 46% in the population; and is 40% among the subpopulation with no ApoE ε4 alleles, and 82% in the subpopulation with at least one ApoE ε4 alleles. Frequency of nuisance disease within the clinical diagnosis varies by ApoE4 status pr(D = 1*|DCL = 1,ε4−) = 0.36 and pr(D = 1*|DCL = 1,ε4+) = 0.06. (DOCX) [file pone.0201140.s002.docx]

| Parameters | True value | Clinical disease status is used as the outcome | | | | With consideration of clinical-pathological relationship | | |
| --- | --- | --- | --- | --- | --- | --- | --- | --- |
|  |  | Usual logistic  regression | | Pseudo-likelihood method  (pMLE) | | Pseudo-likelihood method  (pMLE-DX) | | |
|  |  | Bias | RMSE | Bias | RMSE | Bias | | RMSE |
| $n_{0}=1,000$and $n_{1}=1,000$ | | | | | | | | |
| $\beta_{0}$ | -1 | 0.44 | 0.45 | 0.92 | 0.93 | -0.003 | | 0.11 |
| $\beta_{G}$ | 1.099 | -0.11 | 0.20 | -0.11 | 0.19 | 0.01 | | 0.14 |
| $\beta_{Z_{1}}$ | 1.098 | -0.33 | 0.34 | -0.33 | 0.34 | 0.01 | | 0.14 |
| $\beta_{Z_{2}}$ | -0.083 | 0.03 | 0.10 | 0.03 | 0.09 | -0.003 | | 0.13 |
| $\beta_{\varepsilon4}$ | 2.079 | -0.25 | 0.31 | -0.25 | 0.31 | 0.03 | | 0.22 |
| $\beta_{G\times\varepsilon4}$ | 0 | 0.71 | 3.0 | 0.33 | 1.2 | 0.23 | | 1.3 |
| Pr(G=1) | 0.10 |  |  | -0.0005 | 0.007 | 0.024 | | 0.025 |
| $n_{0}=3,000$and $n_{1}=3,000$ | | | | | | | | |
| $\beta_{0}$ | -1 | 0.45 | 0.45 | 0.93 | 0.93 | | -0.0004 | 0.07 |
| $\beta_{G}$ | 1.099 | -0.12 | 0.15 | -0.07 | -0.15 | | 0.002 | 0.13 |
| $\beta_{Z_{1}}$ | 1.098 | -0.33 | 0.34 | -0.33 | 0.34 | | 0.001 | 0.08 |
| $\beta_{Z_{2}}$ | -0.083 | 0.02 | 0.06 | 0.02 | 0.06 | | -0.003 | 0.08 |
| $\beta_{\varepsilon4}$ | 2.079 | -0.26 | 0.28 | -0.26 | 0.28 | | 0.007 | 0.12 |
| $\beta_{G\times\varepsilon4}$ | 0 | 0.12 | 0.41 | 0.13 | 0.41 | | 0.04 | 0.43 |
| Pr(G=1) | 0.10 |  |  | -0.000 | 0.004 | | 0.03 | 0.03 |
| $n_{0}=5,000$and $n_{1}=5,000$ | | | | | | | | |
| $\beta_{0}$ | -1 | 0.45 | 0.45 | 0.93 | 0.93 | | 0.000 | 0.05 |
| $\beta_{G}$ | 1.099 | -0.12 | 0.14 | -0.12 | 0.14 | | -0.002 | 0.10 |
| $\beta_{Z_{1}}$ | 1.099 | -0.33 | 0.33 | -0.33 | 0.33 | | 0.004 | 0.06 |
| $\beta_{Z_{2}}$ | -0.083 | 0.02 | 0.05 | 0.02 | 0.5 | | -0.005 | 0.06 |
| $\beta_{\varepsilon4}$ | 2.079 | -0.26 | 0.27 | -0.26 | 0.27 | | 0.006 | 0.09 |
| $\beta_{G\times\varepsilon4}$ | 0 | 0.11 | 0.32 | 0.11 | 0.32 | | 0.02 | 0.33 |
| Pr(G=1) | 0.10 |  |  | 0.000 | 0.003 | | 0.03 | 0.03 |
| $n_{0}=10,000$and $n_{1}=10,000$ | | | | | | | | |
| $\beta_{0}$ | -1 | 0.45 | 0.45 | 0.93 | 0.93 | | 0.002 | 0.04 |
| $\beta_{G}$ | 1.099 | -0.12 | 0.13 | -0.12 | 0.13 | | -0.001 | 0.07 |
| $\beta_{Z_{1}}$ | 1.099 | -0.33 | 0.33 | -0.33 | 0.33 | | 0.002 | 0.04 |
| $\beta_{Z_{2}}$ | -0.083 | 0.02 | 0.04 | 0.02 | 0.04 | | -0.006 | 0.04 |
| $\beta_{\varepsilon4}$ | 2.079 | -0.27 | 0.27 | -0.27 | 0.27 | | 0.003 | 0.06 |
| $\beta_{G\times\varepsilon4}$ | 0 | 0.09 | 0.23 | 0.09 | 0.23 | | -0.0006 | 0.24 |
| Pr(G=1) | 0.10 |  |  | 0.000 | 0.002 | | 0.02 | 0.02 |
| $n_{0}=50,000$and $n_{1}=50,000$ | | | | | | | | |
| $\beta_{0}$ | -1 | 0.45 | 0.45 | 0.93 | 0.93 | | 0.0006 | 0.02 |
| $\beta_{G}$ | 1.099 | -0.12 | 0.12 | -0.12 | 0.12 | | 0.0006 | 0.03 |
| $\beta_{Z_{1}}$ | 1.099 | -0.33 | 0.33 | -0.33 | 0.33 | | 0.000 | 0.02 |
| $\beta_{Z_{2}}$ | -0.083 | 0.03 | 0.03 | 0.03 | 0.03 | | -0.001 | 0.02 |
| $\beta_{\varepsilon4}$ | 2.079 | -0.27 | 0.27 | -0.27 | 0.27 | | 0.0006 | 0.02 |
| $\beta_{G\times\varepsilon4}$ | 0 | 0.09 | 0.13 | 0.09 | 0.13 | | -0.002 | 0.10 |
| Pr(G=1) | 0.10 |  |  | 0.000 | 0.001 | | 0.02 | 0.02 |

**S2 Table**. $\boldsymbol{\beta}_{\boldsymbol{G\times\varepsilon}\boldsymbol{4}}\boldsymbol{=}\boldsymbol{0}$. The Bias and Root Mean Squared Error (RMSE) in parameter estimatesfrom simulations using the usual logistic regression with clinical diagnosis as the outcome (uLR), the pseudo-likelihood approach (pMLE), and our newly proposed pseudo-likelihood approach that accounts for misdiagnosis (pMLE-DX). For these simulations, the study included $n_{0}$ controls and $n_{1}$ cases. Frequency of ApoE $\varepsilon$4 allele in the population is 14%. Variables $Z_{1}$ and $Z_{2}$ are Bernoulli with frequencies 0.50 and 0.52, respectively. Frequency of the *true* disease status is 46% in the population; and is 40% among the subpopulation with no ApoE $\varepsilon$4 alleles, and 82% in the subpopulation with at least one ApoE $\varepsilon$4 alleles. Frequency of nuisance disease within the clinical diagnosis varies by ApoE4 status pr(D=$1^{*}|D^{CL}=1,\varepsilon4-$)=0.36 and pr(D=$1^{*}|D^{CL}=1,\varepsilon4+$)=0.06.
