# Supplementary material for: Case-control studies of gene-environment interactions. When a case might not be the case
Source: PLoS One. 2018 Aug 22;13(8):e0201140. doi: 10.1371/journal.pone.0201140 (PMC6104951; doi:10.1371/journal.pone.0201140)
Supplement: S3 Table — The Bias and Root Mean Squared Error (RMSE) in parameter estimates from simulations using the usual logistic regression with clinical diagnosis as the outcome (uLR), the pseudo-likelihood approach (pMLE), and our newly proposed pseudo-likelihood approach that accounts for misdiagnosis (pMLE-DX). For these simulations, the study included n0 = 3000 controls and n1 = 3000 cases. Frequency of ApoE ε4 allele in the population is 14%. Variables Z1 and Z2 are Bernoulli with frequencies 0.50 and 0.52, respectively. Frequency of the true disease status is 46% in the population; and is 40% among the subpopulation with no ApoE ε4 alleles, and 82% in the subpopulation with at least one ApoE ε4 alleles. Frequency of nuisance disease within the clinical diagnosis varies by ApoE4 status pr(D = 1*|DCL = 1,ε4−) = 0.36 and pr(D = 1*|DCL = 1,ε4+) = 0.06. The clinical-pathophysiological diagnoses relationship is misspecified to be pr(D = 1*|DCL = 1,ε4−) = 0.30 and pr(D = 1*|DCL = 1,ε4+) = 0. (DOCX) [file pone.0201140.s003.docx]

| Parameters | True value | With consideration of clinical-pathological diagnoses relationship | | | |
| --- | --- | --- | --- | --- | --- |
|  |  | Pseudolikelihood method (pMLE-DX) | | | |
|  |  | Bias | | RMSE | |
| $n_{0}=1,000$and $n_{1}=1,000$ | | | | | |
| $\beta_{0}$ | -1 | 0.25 | | 0.26 | |
| $\beta_{G}$ | 0.406 | -0.11 | | 0.21 | |
| $\beta_{Z_{1}}$ | 1.098 | -0.26 | | 0.28 | |
| $\beta_{Z_{2}}$ | -0.083 | 0.02 | | 0.11 | |
| $\beta_{\varepsilon4}$ | 2.079 | -0.65 | | 0.68 | |
| $\beta_{G\times\varepsilon4}$ | 0.41 | 1.0 | | 2.2 | |
| Pr(G=1) | 0.10 | 0.02 | | 0.02 | |
| $n_{0}=3,000$and $n_{1}=3,000$ | | | | | |
| $\beta_{0}$ | -1 | 0.25 | 0.02 | | |
| $\beta_{G}$ | 0.406 | -0.10 | 0.16 | | |
| $\beta_{Z_{1}}$ | 1.098 | -0.27 | 0.28 | | |
| $\beta_{Z_{2}}$ | -0.083 | 0.02 | 0.07 | | |
| $\beta_{\varepsilon4}$ | 2.079 | -0.66 | 0.67 | | |
| $\beta_{G\times\varepsilon4}$ | 0.693 | 0.32 | 0.94 | | |
| Pr(G=1) | 0.10 | 0.02 | 0.02 | | |
| $n_{0}=5,000$and $n_{1}=5,000$ | | | | | |
| $\beta_{0}$ | -1 | 0.64 | 0.87 | | |
| $\beta_{G}$ | 0.406 | -0.11 | 0.14 | | |
| $\beta_{Z_{1}}$ | 1.098 | -0.29 | 0.29 | | |
| $\beta_{Z_{2}}$ | -0.083 | 0.02 | 0.05 | | |
| $\beta_{\varepsilon4}$ | 2.079 | -0.66 | 0.66 | | |
| $\beta_{G\times\varepsilon4}$ | 0.406 | 0.20 | 0.55 | | |
| Pr(G=1) | 0.10 | -0.001 | 0.005 | | |
| $n_{0}=10,000$and $n_{1}=10,000$ | | | | | |
| $\beta_{0}$ | -1 | 0.25 | 0.25 | | |
| $\beta_{G}$ | 0.406 | -0.10 | 0.11 | | |
| $\beta_{Z_{1}}$ | 1.098 | -0.27 | 0.27 | | |
| $\beta_{Z_{2}}$ | -0.083 | 0.02 | 0.04 | | |
| $\beta_{\varepsilon4}$ | 2.079 | -0.66 | 0.66 | | |
| $\beta_{G\times\varepsilon4}$ | 0.406 | 0.15 | 0.35 | | |
| Pr(G=1) | 0.10 | 0.03 | 0.03 | | |
| $n_{0}=50,000$and $n_{1}=50,000$ | | | | | |
| $\beta_{0}$ | -1 | 0.25 | 0.03 | |  |
| $\beta_{G}$ | 0.406 | -0.10 | 0.10 | |  |
| $\beta_{Z_{1}}$ | 1.098 | -0.27 | 0.27 | |  |
| $\beta_{Z_{2}}$ | -0.083 | 0.02 | 0.03 | |  |
| $\beta_{\varepsilon4}$ | 2.079 | -0.66 | 0.66 | |  |
| $\beta_{G\times\varepsilon4}$ | 0.406 | 0.12 | 0.18 | |  |
| Pr(G=1) | 0.10 | 0.03 | 0.03 | |  |

**S3 Table**. **Frequency of the nuisance disease is underestimated.** The Bias and Root Mean Squared Error (RMSE) in parameter estimatesfrom simulations using the usual logistic regression with clinical diagnosis as the outcome (uLR), the pseudo-likelihood approach (pMLE), and our newly proposed pseudo-likelihood approach that accounts for misdiagnosis (pMLE-DX). For these simulations, the study included $n_{0}$ = 3000 controls and $n_{1}$ = 3000 cases. Frequency of ApoE $\varepsilon$4 allele in the population is 14%. Variables $Z_{1}$ and $Z_{2}$ are Bernoulli with frequencies 0.50 and 0.52, respectively. Frequency of the *true* disease status is 46% in the population; and is 40% among the subpopulation with no ApoE $\varepsilon$4 alleles, and 82% in the subpopulation with at least one ApoE $\varepsilon$4 alleles. Frequency of nuisance disease within the clinical diagnosis varies by ApoE4 status pr(D=$1^{*}|D^{CL}=1,\varepsilon4-$)=0.36 and pr(D=$1^{*}|D^{CL}=1,\varepsilon4+$)=0.06. The clinical-pathophysiological diagnoses relationship is misspecified to be pr(D=$1^{*}|D^{Cl}=1,\varepsilon4-$)=0.30 and pr(D=$1^{*}|D^{Cl}=1,\varepsilon4+$)=0.
