# Supplementary material for: Case-control studies of gene-environment interactions. When a case might not be the case
Source: PLoS One. 2018 Aug 22;13(8):e0201140. doi: 10.1371/journal.pone.0201140 (PMC6104951; doi:10.1371/journal.pone.0201140)
Supplement: S4 Table — Bias and Root Mean Squared Error (RMSE) for parameter estimates based on a study of 500 simulated datasets with n0 controls and n1 cases with clinical phenotype. Analyses are based on the usual logistic regression model that ignores nuisance disease and based on pseudolikelihood with (pMLE-DX) and without the consideration of clinical-pathological diagnoses relationship (pMLE). Frequency of ApoE ε4 alleles is 14% in the population. Variables Z1 and Z2 are Bernoulli with frequencies 0.50 and 0.52, respectively. Frequency of the true disease status is 46% in the population; and is 40% among the subpopulation with no ApoE ε4 alleles, and 82% in the subpopulation with at least one ApoE ε4 alleles. Frequency of nuisance disease within the clinical diagnosis varies by ApoE4 status pr(D = 1′|DCL = 1,ε4−) = 0.36 and pr(D = 1′|DCL = 1,ε4+) = 0.06. The clinical-pathological diagnoses relationship is misspecified to be pr(D = 1′|DCL = 1,ε4−) = 0.42 and pr(D = 1′|DCL = 1,ε4+) = 0.12. (DOCX) [file pone.0201140.s004.docx]

| Parameters | True value | With consideration of clinical-pathological diagnoses relationship | | | |
| --- | --- | --- | --- | --- | --- |
|  |  | Pseudolikelihood method (pMLE-DX) | | | |
|  |  | Bias | | RMSE | |
| $n_{0}=1,000$and $n_{1}=1,000$ | | | | | |
| $\beta_{0}$ | -1 | -0.18 | | 0.24 | |
| $\beta_{G}$ | 0.406 | 0.04 | | 0.27 | |
| $\beta_{Z_{1}}$ | 1.098 | 0.19 | | 0.26 | |
| $\beta_{Z_{2}}$ | -0.083 | -0.02 | | 0.16 | |
| $\beta_{\varepsilon4}$ | 2.079 | 0.45 | | 0.50 | |
| $\beta_{G\times\varepsilon4}$ | 0.41 | 0.92 | | 2.2 | |
| Pr(G=1) | 0.10 | 0.02 | | 0.02 | |
| $n_{0}=3,000$and $n_{1}=3,000$ | | | | | |
| $\beta_{0}$ | -1 | -0.17 | 0.19 | | |
| $\beta_{G}$ | 0.406 | 0.05 | 0.16 | | |
| $\beta_{Z_{1}}$ | 1.098 | 0.17 | 0.20 | | |
| $\beta_{Z_{2}}$ | -0.083 | -0.02 | 0.09 | | |
| $\beta_{\varepsilon4}$ | 2.079 | 0.43 | 0.44 | | |
| $\beta_{G\times\varepsilon4}$ | 0.693 | 0.17 | 0.93 | | |
| Pr(G=1) | 0.10 | 0.02 | 0.93 | | |
| $n_{0}=5,000$and $n_{1}=5,000$ | | | | | |
| $\beta_{0}$ | -1 | -0.17 | 0.18 | | |
| $\beta_{G}$ | 0.406 | 0.05 | 0.13 | | |
| $\beta_{Z_{1}}$ | 1.098 | 0.17 | 0.19 | | |
| $\beta_{Z_{2}}$ | -0.083 | -0.01 | 0.07 | | |
| $\beta_{\varepsilon4}$ | 2.079 | 0.42 | 0.42 | | |
| $\beta_{G\times\varepsilon4}$ | 0.406 | 0.04 | 0.53 | | |
| Pr(G=1) | 0.10 | 0.02 | 0.02 | | |
| $n_{0}=10,000$and $n_{1}=10,000$ | | | | | |
| $\beta_{0}$ | -1 | -0.17 | 0.18 | | |
| $\beta_{G}$ | 0.406 | 0.06 | 0.10 | | |
| $\beta_{Z_{1}}$ | 1.098 | 0.17 | 0.18 | | |
| $\beta_{Z_{2}}$ | -0.083 | -0.01 | 0.05 | | |
| $\beta_{\varepsilon4}$ | 2.079 | 0.42 | 0.43 | | |
| $\beta_{G\times\varepsilon4}$ | 0.406 | -0.02 | 0.32 | | |
| Pr(G=1) | 0.10 | 0.02 | 0.02 | | |
| $n_{0}=50,000$and $n_{1}=50,000$ | | | | | |
| $\beta_{0}$ | -1 | -0.17 | 0.17 | |  |
| $\beta_{G}$ | 0.406 | 0.06 | 0.07 | |  |
| $\beta_{Z_{1}}$ | 1.098 | 0.17 | 0.17 | |  |
| $\beta_{Z_{2}}$ | -0.083 | -0.01 | 0.02 | |  |
| $\beta_{\varepsilon4}$ | 2.079 | 0.42 | 0.42 | |  |
| $\beta_{G\times\varepsilon4}$ | 0.406 | -0.04 | 0.15 | |  |
| Pr(G=1) | 0.10 | 0.03 | 0.03 | |  |

**S4 Table**. **Frequency of the nuisance disease is overestimated.** Bias and Root Mean Squared Error (RMSE) for parameter estimates based on a study of 500 simulated datasets with $n_{0}$ controls and $n_{1}$ cases with clinical phenotype. Analyses are based on the usual logistic regression model that ignores nuisance disease and based on pseudolikelihood with (pMLE-DX) and without the consideration of clinical-pathological diagnoses relationship (pMLE). Frequency of ApoE $\varepsilon$4 alleles is 14% in the population. Variables $Z_{1}$ and $Z_{2}$ are Bernoulli with frequencies 0.50 and 0.52, respectively. Frequency of the *true* disease status is 46% in the population; and is 40% among the subpopulation with no ApoE $\varepsilon$4 alleles, and 82% in the subpopulation with at least one ApoE $\varepsilon$4 alleles. Frequency of nuisance disease within the clinical diagnosis varies by ApoE4 status pr(D=$1^{'}|D^{Cl}=1,\varepsilon4-$)=0.36 and pr(D=$1^{'}|D^{Cl}=1,\varepsilon4+$)=0.06. The clinical-pathological diagnoses relationship is misspecified to be pr(D=$1^{'}|D^{Cl}=1,\varepsilon4-$)=0.42 and pr(D=$1^{'}|D^{Cl}=1,\varepsilon4+$)=0.12.
