# Supplementary material for: Case-control studies of gene-environment interactions. When a case might not be the case
Source: PLoS One. 2018 Aug 22;13(8):e0201140. doi: 10.1371/journal.pone.0201140 (PMC6104951; doi:10.1371/journal.pone.0201140)
Supplement: S6 Table — The Bias and Root Mean Squared Error (RMSE) in parameter estimates from simulations using the usual logistic regression with clinical diagnosis as the outcome (uLR), the pseudo-likelihood approach (pMLE), and our newly proposed pseudo-likelihood approach that accounts for misdiagnosis (pMLE-DX). For these simulations, the study included n0 controls and n1 cases. Risk of the disease of interest is defined in a set of parameters β0,βG,βZ1,βZ2,βG×ε4; while the risk of the nuisance disease is parametrized by β0*,βG*,βε4*,βG×ε4*. Frequency of ApoE ε4 allele in the population is 14%. Variables Z1 and Z2 are Bernoulli with frequencies 0.50 and 0.52, respectively. Frequencies of the disease of interest and the nuisance disease are pr(D = 1) = 24.8%, pr(D = 1*) = 12.5%, pr(D = 1|ε4+) = 43%, pr(D = 1*|ApoE4+) = 16.1%, pr(D = 1|ε4−) = 20%, pr(D = 1*|ε4−) = 11.6%. Frequency of the nuisance disease within the clinical diagnosis varies by ApoE4 status pr(D = 1*|DCL = 1,ε4−) = 0.36 and pr(D = 1*|DCL = 1,ε4+) = 0.06. (DOCX) [file pone.0201140.s006.docx]

| Parameters | True value | Clinical disease status is used as the outcome | | | | With consideration of clinical-pathological diagnoses relationship | | |
| --- | --- | --- | --- | --- | --- | --- | --- | --- |
|  |  | Usual logistic  regression | | Pseudo-likelihood method  (pMLE) | | Pseudo-likelihood method  (pMLE-DX) | | |
|  |  | Bias | RMSE | Bias | RMSE | Bias | | RMSE |
| $n_{0}=1,000$and $n_{1}=1,000$ | | | | | | | | |
| $\beta_{0}$ | -1 | 0.97 | 0.97 | 0.75 | 0.75 | 0.03 | | 0.10 |
| $\beta_{0}^{*}$ | -1.7 |  |  |  |  | 0.03 | | 0.10 |
| $\beta_{G}$ | -0.69 | 0.29 | 0.34 | -0.39 | 0.42 | -0.004 | | 0.16 |
| $\beta_{G}^{*}$ | 0 |  |  |  |  | -0.03 | | 0.24 |
| $\beta_{Z_{1}}$ | 0.10 | 0.005 | 0.10 | 0.004 | 0.10 | 0.005 | | 0.10 |
| $\beta_{Z_{2}}$ | -0.083 | -0.002 | 0.09 | -0.0007 | 0.09 | -0.002 | | 0.09 |
| $\beta_{\varepsilon4}$ | 1.3 | -0.21 | 0.25 | -0.21 | 0.25 | 0.002 | | 0.18 |
| $\beta_{\varepsilon4}^{*}$ | 0.5 |  |  |  |  | -0.01 | | 0.14 |
| $\beta_{G\times\varepsilon4}$ | 0 | -0.25 | 0.55 | -0.25 | 0.55 | -0.002 | | 0.45 |
| $\beta_{G\times\varepsilon4}^{*}$ | 0 |  |  |  |  | -0.005 | | 0.25 |
| Pr(G=1) | 0.10 |  |  | 0.05 | 0.007 | 0.000 | | 0.007 |
| $n_{0}=5,000$and $n_{1}=5,000$ | | | | | | | | |
| $\beta_{0}$ | -1 | 0.97 | 0.97 | 0.75 | 0.75 | | 0.03 | 0.05 |
| $\beta_{0}^{*}$ | -1.7 |  |  |  |  | | 0.01 | 0.05 |
| $\beta_{G}$ | -0.69 | 0.30 | 0.31 | -0.38 | 0.39 | | 0.003 | 0.08 |
| $\beta_{G}^{*}$ | 0 |  |  |  |  | | 0.003 | 0.10 |
| $\beta_{Z_{1}}$ | 0.10 | -0.001 | 0.04 | -0.002 | 0.04 | | -0.001 | 0.04 |
| $\beta_{Z_{2}}$ | -0.083 | -0.004 | 0.04 | -0.004 | 0.04 | | -0.004 | 0.04 |
| $\beta_{\varepsilon4}$ | 1.3 | -0.22 | 0.23 | -0.22 | 0.23 | | -0.005 | 0.08 |
| $\beta_{\varepsilon4}^{*}$ | 0.5 |  |  |  |  | | -0.006 | 0.08 |
| $\beta_{G\times\varepsilon4}$ | 0 | -0.23 | 0.28 | -0.23 | 0.38 | | 0.01 | 0.19 |
| $\beta_{G\times\varepsilon4}^{*}$ | 0 |  |  |  |  | | 0.0003 | 0.10 |
| Pr(G=1) | 0.10 |  |  | 0.05 | 0.003 | | 0.000 | 0.003 |
| $n_{0}=10,000$and $n_{1}=10,000$ | | | | | | | | |
| $\beta_{0}$ | -1 | 0.97 | 0.97 | 0.75 | 0.75 | | 0.03 | 0.002 |
| $\beta_{0}^{*}$ | -1.7 |  |  |  |  | | 0.02 | 0.04 |
| $\beta_{G}$ | -0.69 | 0.30 | 0.31 | -0.38 | 0.38 | | -0.0002 | 0.05 |
| $\beta_{G}^{*}$ | 0 |  |  |  |  | | -0.0006 | 0.08 |
| $\beta_{Z_{1}}$ | 0.10 | 0.000 | 0.03 | -0.0006 | 0.03 | | 0.000 | 0.03 |
| $\beta_{Z_{2}}$ | -0.083 | -0.003 | 0.03 | -0.002 | 0.03 | | -0.003 | 0.03 |
| $\beta_{\varepsilon4}$ | 1.3 | -0.22 | 0.22 | -0.21 | 0.22 | | -0.0007 | 0.06 |
| $\beta_{\varepsilon4}^{*}$ | 0.5 |  |  |  |  | | -0.004 | 0.03 |
| $\beta_{G\times\varepsilon4}$ | 0 | -0.22 | 0.38 | -0.24 | 0.37 | | -0.001 | 0.13 |
| $\beta_{G\times\varepsilon4}^{*}$ | 0 |  |  |  |  | | -0.004 | 0.07 |
| Pr(G=1) | 0.10 |  |  | 0.05 | 0.002 | | 0.000 | 0.002 |
| $n_{0}=50,000$and $n_{1}=50,000$ | | | | | | | | |
| $\beta_{0}$ | -1 | 0.97 | 0.97 | 0.75 | 0.75 | | 0.03 | 0.03 |
| $\beta_{0}^{*}$ | -1.7 |  |  |  |  | | 0.02 | 0.02 |
| $\beta_{G}$ | -0.69 | 0.30 | 0.30 | -0.38 | 0.38 | | -0.001 | 0.02 |
| $\beta_{G}^{*}$ | 0 |  |  |  |  | | 0.001 | 0.03 |
| $\beta_{Z_{1}}$ | 0.10 | 0.0004 | 0.01 | -0.0003 | 0.01 | | 0.0004 | 0.01 |
| $\beta_{Z_{2}}$ | -0.083 | 0.000 | 0.01 | 0.0005 | 0.01 | | 0.000 | 0.01 |
| $\beta_{\varepsilon4}$ | 1.3 | -0.22 | 0.22 | -0.21 | 0.21 | | 0.0002 | 0.02 |
| $\beta_{\varepsilon4}^{*}$ | 0.5 |  |  |  |  | | -0.001 | 0.02 |
| $\beta_{G\times\varepsilon4}$ | 0 | -0.35 | 0.36 | -0.35 | 0.35 | | -0.005 | 0.06 |
| $\beta_{G\times\varepsilon4}^{*}$ | 0 |  |  |  |  | | -0.003 | 0.03 |
| Pr(G=1) | 0.10 |  |  |  |  | | -0.0002 | 0.001 |

**S6 Table**. $\boldsymbol{\beta}_{\boldsymbol{G\times\varepsilon}\boldsymbol{4}}\boldsymbol{=0,}\boldsymbol{\beta}_{\boldsymbol{G\times}\boldsymbol{\varepsilon}\boldsymbol{4}}^{\boldsymbol{*}}\boldsymbol{=0,}\boldsymbol{\beta}_{\boldsymbol{G}}^{\boldsymbol{*}}\boldsymbol{=0.}$ The Bias and Root Mean Squared Error (RMSE) in parameter estimatesfrom simulations using the usual logistic regression with clinical diagnosis as the outcome (uLR), the pseudo-likelihood approach (pMLE), and our newly proposed pseudo-likelihood approach that accounts for misdiagnosis (pMLE-DX). For these simulations, the study included $n_{0}$controls and $n_{1}$ cases. Risk of the disease of interest is defined in a set of parameters $\beta_{0}, \beta_{G}, \beta_{Z_{1}},\beta_{Z_{2}}, \beta_{G\times\varepsilon4}$; while the risk of the nuisance disease is parametrized by $\beta_{0}^{*}, \beta_{G}^{*}, \beta_{\varepsilon4}^{*}, \beta_{G\times\varepsilon4}^{*}.$ Frequency of ApoE $\varepsilon$4 allele in the population is 14%. Variables $Z_{1}$ and $Z_{2}$ are Bernoulli with frequencies 0.50 and 0.52, respectively. Frequencies of the disease of interest and the nuisance disease are pr(D=1)=24.8%, pr(D=$1^{*})=12.5\%, \mathrm{pr}\left( D=1 | \varepsilon4+ \right)=43\%, \mathrm{pr}\left( D=1^{*} | ApoE4+ \right)=16.1\%, \mathrm{pr}\left( D=1 | \varepsilon4- \right)=20\%, \mathrm{pr}\left( D=1^{*} | \varepsilon4- \right)=11.6\%.$ Frequency of the nuisance disease within the clinical diagnosis varies by ApoE4 status pr(D=$1^{*}|D^{CL}=1,\varepsilon4-$)=0.36 and pr(D=$1^{*}|D^{CL}=1,\varepsilon4+$)=0.06.
