# Supplementary material for: Case-control studies of gene-environment interactions. When a case might not be the case
Source: PLoS One. 2018 Aug 22;13(8):e0201140. doi: 10.1371/journal.pone.0201140 (PMC6104951; doi:10.1371/journal.pone.0201140)
Supplement: S7 Table — Analyses are performed using the usual logistic regression (uLR) that uses the clinical diagnosis as an outcome and using pseudo-likelihood method that assumes that the proportion of nuisance disease within the clinically diagnosed AD is 36% for ε4 carriers and is 6% for ε4 non-carriers. Pseudo-likelihood analyses pMLE-DX estimates parameters for D = 1 vs. D = 0 and D = 1* combined. Pseudo-likelihood analyses pMLE − DX*, however, estimate two sets of risk coefficients, i.e. βs for D = 0 vs. D = 1 and β*s D = 0 vs. D = 1*. (DOCX) [file pone.0201140.s007.docx]

| **SNP** | $\hat{\boldsymbol{\beta}_{\boldsymbol{\varepsilon}\boldsymbol{4}}^{\boldsymbol{*}}}$ | $\hat{\boldsymbol{\beta}_{\boldsymbol{G}}^{\boldsymbol{*}}}$ | $\hat{\boldsymbol{\beta}_{\boldsymbol{G\times\varepsilon}\boldsymbol{4}}^{\boldsymbol{*}}}$ |
| --- | --- | --- | --- |
| **rs2033831** | **1.8**, p=0.02 | 3.8, p=0.47 | -0.50, p=0.64 |
| **rs7656500** | **0.36**, p=0.01 | 5.0, p=0.12 | 0.91, p=0.15 |
| **rs1816702** | **1.2**, p=0.01 | 5.0, p=0.38 | -0.49, p=0.12 |
| **rs830832** | 2.1, p=0.11 | 4.2, p=0.06 | -0.68, p=0.67 |
| **rs7676342** | **1.7**, p=0.02 | 4.1, p=0.48 | -**2.1,** p=0.02 |
| **rs4862611** | 0.70, p=0.23 | 3.4, p=0.25 | 1.0, p=0.10 |
| **rs13113778** | **1.4**, p=0.00 | 5.0, p=0.29 | **-2.7**,p=0.03 |
| **rs1869617** | 0.52, p=0.20 | 5.0, p=0.17 | 0.58, p=0.27 |
| **rs11938703** | **4.4**, p=0.00 | **4.6**, p=0.05 | -1.7, p=0.11 |
| **rs1519318** | **-4.1**, p=0.00 | **2.7**, p=0.01 | 4.9, p=0.31 |
| **rs12648771** | **1.6**, p=0.01 | 5.0, p=0.14 | 0.46, p=0.51 |
| **rs6894** | 0.21, p=0.59 | **5.0**, p=0.05 | 0.37, p=0.28 |
| **rs3775296** | **1.6**, p=0.02 | 4.9, p=0.06 | -0.68, p=0.24 |
| **rs7668666** | 1.0, p=0.07 | 3.1, p=0.056 | 1.1, p=0.81 |
| **rs1706143** | **1.3**, p=0.00 | -0.12, p=0.20 | 0.12,p=0.74 |
| **rs9299251** | **1.1**, p=0.00 | -0.04, p=0.41 | 0.35, p=0.05 |
| **rs955302** | **-2.1**, p=0.026 | 2.7, p=0.11 | **4.0**, p=0.01 |
| **rs17419570** | **1.6**, p=0.01 | **4.8**, p=0.04 | -0.80, p=0.13 |
| **rs16905625** | **1.7**, p=0.02 | 4.8, p=0.09 | -0.11, p=0.42 |
| **rs10513307** | **1.6**, p=0.00 | 5.0, p=0.08 | 0.20, p=0.21 |
| **rs1890047** | **1.6**, p=0.01 | **4.8**, p=0.04 | -0.80, p=0.13 |
| **rs4837254** | -0.58, p=0.17 | 2.7, p=0.25 | **2.3**, p=0.04 |
| **rs13285674** | 1.8, p=0.12 | 3.4, p=0.15 | -0.05, p=0.32 |
| **rs1337208** | 1.9, p=0.07 | 4.2, p=0.19 | -0.46, p=0.32 |
| **rs1415378** | **3.1**, p=0.01 | **5.0**, p=0.01 | -1.9, p=0.6 |
| **rs504204** | **0.04**, p=0.00 | **5.0**, p=0.04 | 3.4, p=0.38 |
| **rs12337381** | **2.9**, p=0.00 | 5.0, p=0.07 | -0.53, p=0.054 |
| **rs1952464** | 1.3, p=0.59 | 3.7, p=0.17 | 1.4, p=0.09 |
| **rs12342331** | -0.41, p=0.41 | **5.0**, p=0.03 | **2.1**, p=0.04 |
| **rs16905754** | 1.2, p=0.11 | 5.0, p=0.16 | 2.7, p=0.60 |
| **rs2771054** | **0.50**, p=0.01 | 5.0, p=0.58 | 0.68, p=0.22 |
| **rs521581** | **-1.1**, p=0.00 | -0.02, p=0.46 | **0.83**, p=0.044 |
| **rs1329063** | -2.2, p=0.17 | 5.0, p=0.58 | 3.9, p=0.08 |
| **rs495083** | **-3.6**, p=0.02 | 2.6, p=0.32 | **4.8**, p=0.01 |
| **rs476** | **3.0**, p=0.00 | **4.9**, p=0.03 | -1.3, p=0.11 |
| **rs565055** | 1.5, p=0.08 | 3.3, p=0.40 | -0.53, p=0.38 |
| **rs2094630** | 3.1,p=0.13 | 4.5, p=0.52 | -1.4, p=0.18 |
| **rs10983712** | **-3.0**, p=0.01 | **4.9**, p=0.02 | **4.9**, p=0.001 |
| **rs10983736** | -0.91, p=0.12 | 5, p=0.068 | 0.45, p=0.27 |
| **rs16905962** | -1.4, p=0.15 | 5.0, p=0.67 | 2.6, p=0.22 |
| **rs1927914** | 1.1, p=0.12 | 3.0, p=0.33 | 1.2, p=0.08 |
| **rs11536879** | -0.95, p=0.17 | 5.0, p=0.27 | **4.0**, p=0.02 |
| **rs4986790** | -1.6, p=0.11 | 5.0, p=0.19 | 3.1, p=0.10 |
| **rs7045953** | **-2.8**, p=0.016 | 4.8, p=0.40 | **2.5**, p=0.04 |
| **rs7357627** | **3.2**, p=0.01 | **4.9**, p=0.00 | **-3.2**, p=0.02 |
| **rs7046020** | **1.4**, p=0.03 | 3.3, p=0.28 | 0.38, p=0.60 |
| **rs1927937** | 1.1, p=0.11 | **1.3**, p=0.02 | 2.7, p=0.05 |
| **rs1927924** | **1.8**, p=0.01 | 5.0, p=0.76 | -0.88, p=0.07 |
| **rs3860141** | 1.2, p=0.15 | 2.5, p=0.20 | 1.0, p=0.13 |
| **rs1877876** | **3.0**, p=0.00 | 3.9, p=0.54 | -0.62, p=0.15 |
| **rs497322** | **1.7**, p=0.01 | 5.0, p=0.052 | 0.34, p=0.58 |
| **rs6478330** | **0.59**, p=0.001 | 5.0, p=0.41 | 0.66, p=0.20 |
| **rs7856175** | **1.4**, p=0.04 | 2.3, p=0.34 | 0.61, p=0.33 |
| **rs3134940** | 0.14, p=0.99 | **2.0**, p=0.01 | 0.54, p=0.89 |
| **rs1035798** | 0.14, p=0.99 | **1.8**, p=0.01 | 0.14, p=0.79 |
| **rs2070600** | **0.61**, p=0.00 | **2.1**, p=0.01 | 0.23, p=0.69 |

**S7 Table**. **Parameter estimates in Alzheimer’s Disease study.** Analyses are performed using the usual logistic regression (uLR) that uses the clinical diagnosis as an outcome and using pseudo-likelihood method that assumes that the proportion of nuisance disease within the clinically diagnosed AD is 36% for $\varepsilon4$carriers and is 6% for $\varepsilon4$non-carriers. Pseudo-likelihood analyses pMLE-DX estimates parameters for *D*=1 vs. *D*=0 and *D*=$1^{*}$ combined$.$ Pseudo-likelihood analyses$pMLE-DX^{*}$, however, estimate two sets of risk coefficients, i.e. $\beta$s for$D=0$ vs. $D=1$ and $\beta^{*}s$ $D=0$ vs. $D=1^{*}$.
