# Supplementary material for: Case-control studies of gene-environment interactions. When a case might not be the case
Source: PLoS One. 2018 Aug 22;13(8):e0201140. doi: 10.1371/journal.pone.0201140 (PMC6104951; doi:10.1371/journal.pone.0201140)
Supplement: S8 Table — (Table 5, SNPs whose effect estimates of βG and/or βG×ε4 are with permutation-based p-value <0.05). (DOCX) [file pone.0201140.s008.docx]

| **Alzheimer’s Disease** | | **Vascular Disease** | | **Dementia** | | **Taopathy** | |
| --- | --- | --- | --- | --- | --- | --- | --- |
| SNPs inferred in Alzheimer’s disease study (Table 5) | SNPs previously reported within 500k up- or downstream | SNPs inferred in Alzheimer’s disease study (Table 5) | SNPs previously reported within 500k up- or downstream | SNPs inferred in Alzheimer’s disease study (Table 5) | SNPs previously reported within 500k up- or downstream | SNPs inferred in Alzheimer’s disease study (Table 5) | SNPs previously reported within 500k up- or downstream |
| \| rs2033831 \| \| --- \| \| rs2033831 \| \| rs7656500 \| \| rs7656500 \| \| rs1816702 \| \| rs1816702 \| \| rs830832 \| \| rs7676342 \| \| rs4862611 \| \| rs13113778 \| \| rs1869617 \| \| rs11938703 \| \| rs1519318 \| \| rs12648771 \| \| rs3775296 \| \| rs7668666 \| \| rs1706143 \| \| rs3775296 \| \| rs7668666 \| \| rs1706143 \| | \| rs727153 \| \| --- \| \| rs1466662 \| \| rs727153 \| \| rs1466662 \| \| rs727153 \| \| rs1466662 \| \| rs75718659 \| \| rs75718659 \| \| rs75718659 \| \| rs75718659 \| \| rs75718659 \| \| rs75718659 \| \| rs75718659 \| \| rs75718659 \| \| rs75718659 \| \| rs75718659 \| \| rs75718659 \| \| rs75718659 \| \| rs75718659 \| \| rs75718659 \| | \| rs2033831 \| \| --- \| \| rs2033831 \| \| rs2033831 \| \| rs2033831 \| \| rs2033831 \| \| rs2033831 \| \| rs7656500 \| \| rs7656500 \| \| rs7656500 \| \| rs7656500 \| \| rs7656500 \| \| rs7656500 \| \| rs1816702 \| \| rs1816702 \| \| rs1816702 \| \| rs1816702 \| \| rs1816702 \| \| rs1816702 \| | \| rs7654093 \| \| --- \| \| rs7659024 \| \| rs2066865 \| \| rs2066865 \| \| rs6536024 \| \| rs11099942 \| \| rs7654093 \| \| rs7659024 \| \| rs2066865 \| \| rs2066865 \| \| rs6536024 \| \| rs11099942 \| \| rs7654093 \| \| rs7659024 \| \| rs2066865 \| \| rs2066865 \| \| rs6536024 \| \| rs11099942 \| | \| rs2033831 \| \| --- \| \| rs2033831 \| \| rs7656500 \| \| rs7656500 \| \| rs1816702 \| \| rs1816702 \| \| rs830832 \| \| rs7676342 \| \| rs4862611 \| \| rs13113778 \| \| rs1869617 \| \| rs11938703 \| \| rs1519318 \| \| rs12648771 \| \| rs3775296 \| \| rs7668666 \| \| rs1706143 \| \| rs9299251 \| \| rs955302 \| \| rs17419570 \| \| rs16905625 \| \| rs10513307 \| \| rs1890047 \| \| rs4837254 \| \| rs13285674 \| \| rs1337208 \| \| rs1415378 \| \| rs3775296 \| \| rs7668666 \| \| rs1706143 \| \| rs9299251 \| \| rs955302 \| \| rs17419570 \| \| rs16905625 \| \| rs10513307 \| \| rs1890047 \| \| rs4837254 \| \| rs13285674 \| \| rs1337208 \| \| rs1415378 \| \| rs504204 \| \| rs12337381 \| \| rs1952464 \| \| rs12342331 \| | \| rs727153 \| \| --- \| \| rs1466662 \| \| rs727153 \| \| rs1466662 \| \| rs727153 \| \| rs1466662 \| \| rs75718659 \| \| rs75718659 \| \| rs75718659 \| \| rs75718659 \| \| rs75718659 \| \| rs75718659 \| \| rs75718659 \| \| rs75718659 \| \| rs75718659 \| \| rs75718659 \| \| rs75718659 \| \| rs1360695 \| \| rs1360695 \| \| rs1360695 \| \| rs1360695 \| \| rs1360695 \| \| rs1360695 \| \| rs1360695 \| \| rs1360695 \| \| rs1360695 \| \| rs1360695 \| \| rs75718659 \| \| rs75718659 \| \| rs75718659 \| \| rs1360695 \| \| rs1360695 \| \| rs1360695 \| \| rs1360695 \| \| rs1360695 \| \| rs1360695 \| \| rs1360695 \| \| rs1360695 \| \| rs1360695 \| \| rs1360695 \| \| rs1360695 \| \| rs1360695 \| \| rs1360695 \| \| rs1360695 \| | \| rs2033831 \| \| --- \| \| rs2033831 \| \| rs7656500 \| \| rs7656500 \| \| rs1816702 \| \| rs1816702 \| \| rs830832 \| \| rs7676342 \| \| rs4862611 \| \| rs13113778 \| \| rs1869617 \| \| rs11938703 \| \| rs1519318 \| \| rs12648771 \| \| rs3775296 \| \| rs7668666 \| \| rs1706143 \| \| rs3775296 \| \| rs7668666 \| \| rs1706143 \| | \| rs727153 \| \| --- \| \| rs1466662 \| \| rs727153 \| \| rs1466662 \| \| rs727153 \| \| rs1466662 \| \| rs75718659 \| \| rs75718659 \| \| rs75718659 \| \| rs75718659 \| \| rs75718659 \| \| rs75718659 \| \| rs75718659 \| \| rs75718659 \| \| rs75718659 \| \| rs75718659 \| \| rs75718659 \| \| rs75718659 \| \| rs75718659 \| \| rs75718659 \| |

**S8 Table: SNPs previously reported in GWAS that are within 500k up- or downstream of SNPs that we inferred in Alzheimer’s disease study** (Table 5, SNPs whose effect estimates of $\beta_{G}$ and/or $\beta_{G\times\varepsilon4}$ are with permutation-based p-value <0.05).
